# Supplementary material for: Defect-induced triple synergistic modulation in copper for superior electrochemical ammonia production across broad nitrate concentrations
Source: Nat Commun. 2024 Apr 1;15:2816. doi: 10.1038/s41467-024-47025-w (PMC10984973; doi:10.1038/s41467-024-47025-w)
Supplement: Supplementary file 1 — Supplementary Information [file 41467_2024_47025_MOESM1_ESM.pdf]

Supplementary Information

Defect-Induced Triple Synergistic Modulation in Copper for Superior Electrochemical Ammonia Production across Broad Nitrate Concentrations

*Bocheng Zhang[1+], Zechuan Dai[1+], Yanxu Chen[1+], Mingyu Cheng[1], Huaikun Zhang[1], Pingyi Feng[1], Buqi Ke[1], Yangyang Zhang[1], Genqiang Zhang[1]**

B. C. Zhang, Z. C. Dai, Y. X. Chen, M. Y. Cheng, H. K. Zhang, P. Y. Feng, B. Q. Ke, Y. Y. Zhang, Prof. G. Q. Zhang

Affiliations

[1]Hefei National Research Center for Physical Sciences at the Microscale, CAS Key Laboratory of Materials for Energy Conversion, Department of Materials Science and Engineering, University of Science and Technology of China, Hefei, Anhui 230026, China.

Email: gqzhangmse@ustc.edu.cn

[+]These three authors contribute equally to this work.

1. Supplementary Figures **5**

Supplementary Figure 1. High-resolution images of the morphology of Cu_3N NWs and the lattice of $Cu_3N(100)$.

Supplementary Figure 2. SEM image of $Cu(OH)_2$.

Supplementary Figure 3. XPS spectra of Cu_3N NWs and V-Cu NAE.

Supplementary Figure 4. Survey XPS spectra of Cu_3N NWs and V-Cu NAE.

Supplementary Figure 5. The structure of the in-situ Raman electrolytic cell.

Supplementary Figure 6. XPS spectra of Cu NWs and V-Cu NAE

Supplementary Figure 7. Photograph of the H-cell setup

Supplementary Figure 8. The concentration-absorbance UV-vis calibration curve of NH_4^+, NO_3^-, NO_2^-.

Supplementary Figure 9. Potential-dependent Faradaic efficiency and selectivity of ammonia over Cu_3N NWs and Cu NWs.

Supplementary Figure 10. Current density, FE of NH_3, selectivity of NH_3, and conversion of NO_3^- in Cu NWs under different concentration nitrate sources.

Supplementary Figure 11. The cyclic voltammograms (CV) scan rate-current relationship and corresponding electrochemical active surface area (ECSA) analysis.

Supplementary Figure 12. ^1H NMR spectra before and after NO_3RR using $^{15}NO_3^-$ and $^{14}NO_3^-$ electrolytes.

Supplementary Figure 13. A standard curve was constructed by plotting the integral area of $^{15}NH_4^+$-^{15}N and $^{14}NH_4^+$-^{14}N relative to $C_4H_4O_4$ against the $^{15}NH_4^+$-^{15}N and $^{14}NH_4^+$-^{14}N concentration.

Supplementary Figure 14. Comparison of ammonia yield and Faradaic efficiency obtained by colorimetric method and nuclear magnetic quantification method.

Supplementary Figure 15. Laboratory setup of the synchrotron radiation in-situ infrared apparatus.

Supplementary Figure 16. Photograph of In-situ infrared electrolytic cell.

Supplementary Figure 17. Operando Synchrotron Radiation-FTIR spectroscopy measurements under various potentials for V-Cu NAE during Electrolysis without NO_3^-.

Supplementary Figure 18. Front view, top view, and side view of the Cu (111) surface with four explicit water molecules.

Supplementary Figure 19. Front view, top view, and side view of the V-Cu (111) surface with four explicit water molecules.

Supplementary Figure 20. Constant potential simulation of water splitting and HER. The $E_{free}(U_{SHE})$ of $*H_2O$, *OH+*H systems for Cu and V-Cu.

Supplementary Figure 21. The reaction mechanism of NO_3RR and the intermediates involved.

Supplementary Figure 22. The $E_{free}(U_{SHE})$ of slab, *O, *N, *OH, *NH, *NO, $*NH_2$, *NOH, *NHO, $*NO_2$, $*NH_3$, *NHOH, $*NH_2O$, $*NH_2OH$ and $*NO_3$ systems for Cu and V-Cu.

Supplementary Figure 23. Free energy as a function of the CV of NO_3RR pathway by Slow-growth method for Cu.

Supplementary Figure 24. Free energy as a function of the CV of NO_3RR pathway

by Slow-growth method for V-Cu.
Supplementary Figure 25. The coverage of key intermediates during the reaction, namely *, *NO_3, *NO_2, *O and *OH, as a function of time for Cu and V-Cu. The reaction reaches the steady state, nitrite and ammonia productive rate.
Supplementary Figure 26. The intermediate adsorption structural model for NO_3RR over front view of Cu(111).
Supplementary Figure 27. The intermediate adsorption structural model for NO_3RR over front view of V-Cu(111).
Supplementary Figure 28. The transition state (TS) structural model for NO_3RR over front view of Cu(111).
Supplementary Figure 29. The transition state (TS) structural model for NO_3RR over front view of V-Cu(111).
Supplementary Figure 30. The thermodynamic cycle used to calculate the adsorption Gibbs free energy of NO_3^- in the gas phase.
Supplementary Figure 31. The thermodynamic cycle used to calculate the adsorption Gibbs free energy of NO_2^- in the gas phase.
Supplementary Figure 32. Schematic diagram of the two-electrode flow cell device.
Supplementary Figure 33. A standard curve was generated by plotting the integral area of $HCOO^-$-^{1}H relative to a specific peak of DMSO against the ^{1}H concentration in formate ion.
Supplementary Figure 34. XRD spectra of V-Cu NAE catalyst before and after 120 h electrocatalytic NO_3RR.
Supplementary Figure 35. SEM images of the V-Cu NAE catalyst before and after 120 h electrocatalytic NO_3^-RR.
Supplementary Figure 36. XPS spectra of Cu NWs, V-Cu NAE and V-Cu NAE after 120 hours of cycling.

2. Supplementary Tables 27
Supplementary Table 1. Comparison of the electrocatalytic activities of V-Cu NAE with other reported materials for NO_3RR.
Supplementary Table 2. Calculated Gibbs free energies of NO_3RR at -0.289 V vs RHE and T = 298.15 K for Cu and V-Cu.
Supplementary Table 3. Activation energies of NO_3RR at -0.289 V vs RHE and T = 298.15 K for Cu and V-Cu.
Supplementary Table 4. Parameters for fitting the relationship between E_{free} and U under constant potential simulation for Cu.
Supplementary Table 5. Parameters for fitting the relationship between E_{free} and U under constant potential simulation for V-Cu.
Supplementary Table 6. Parameters used by Slow-growth approach for Cu.
Supplementary Table 7. Parameters used by Slow-growth approach for V-Cu.
Supplementary Table 8. Calculated Gibbs free energies of NO_3RR at -0.289 V vs RHE and T = 298.15 K for Cu and V-Cu.
Supplementary Table 9. Activation energies of NO_3RR at -0.289 V vs RHE and T = 298.15 K for Cu and V-Cu.

3. Supplementary Notes **36**

Supplementary Note S1. Details of Microkinetic Simulations.

Supplementary Note S2. Details of Slow-growth method.

4. References for Supplementary Information **40**

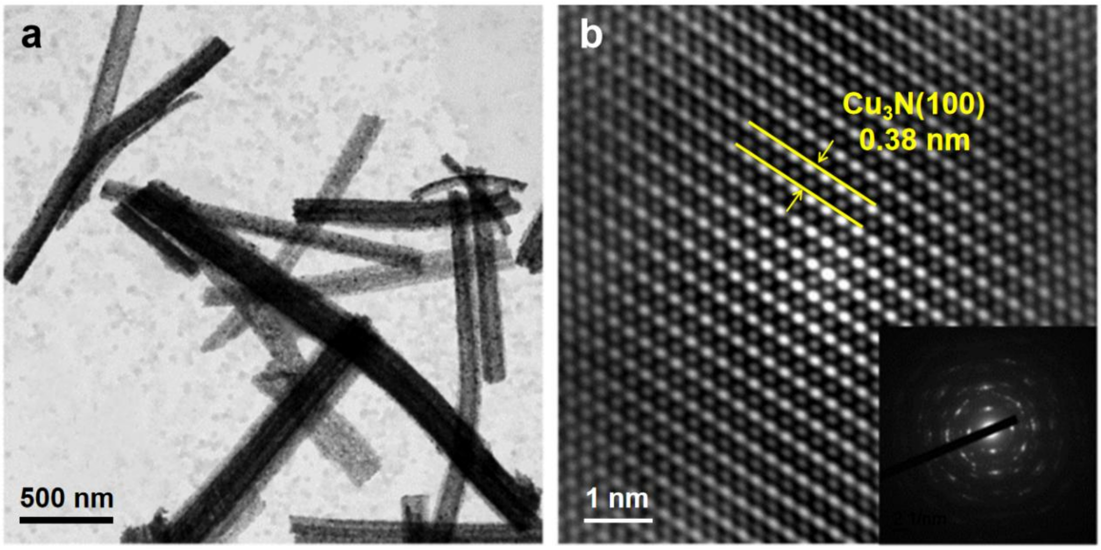

Supplementary Figure 1. **High-Resolution Images of the morphology of Cu_3N NWs and the lattice of Cu_3N (100).** (a) The morphology of Cu_3N NWs. (b) The lattice of Cu_3N (100).

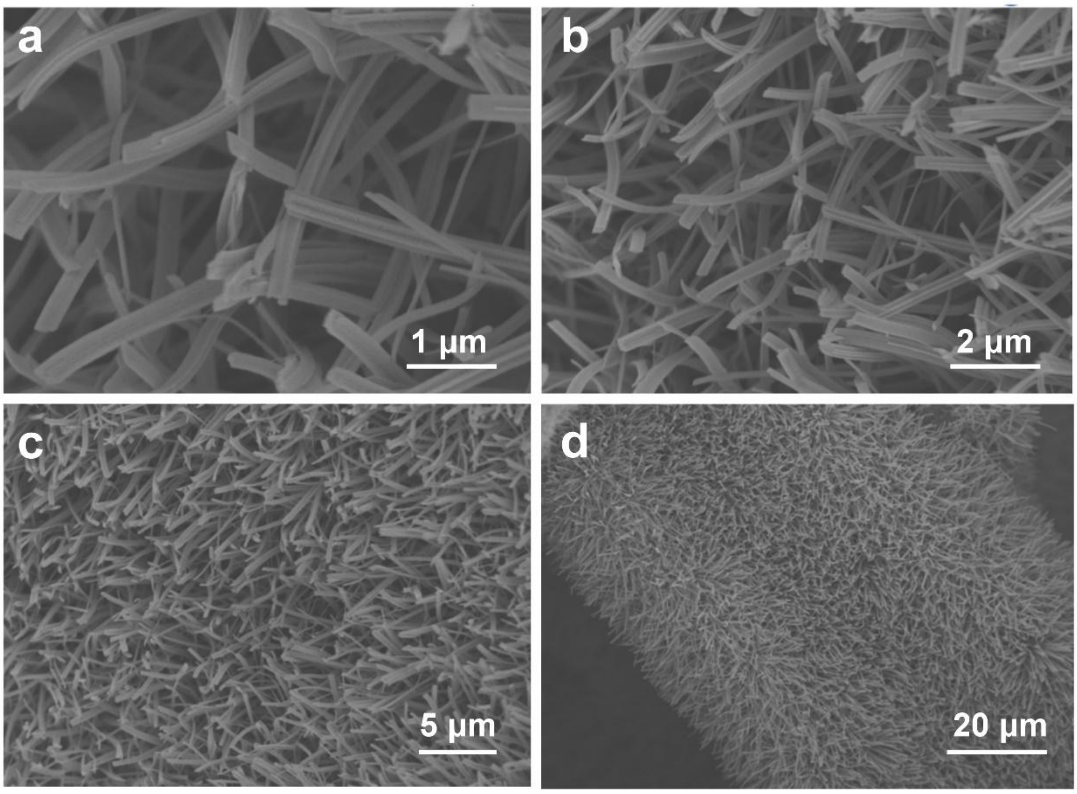

Supplementary Figure 2. **SEM images of $Cu(OH)_2$ NWs.** (a) High-resolution detail, 1 μm scale. (b) Increased area, 2 μm scale. (c) Expanded coverage, 5 μm scale. (d) Wide-field overview, 20 μm scale.

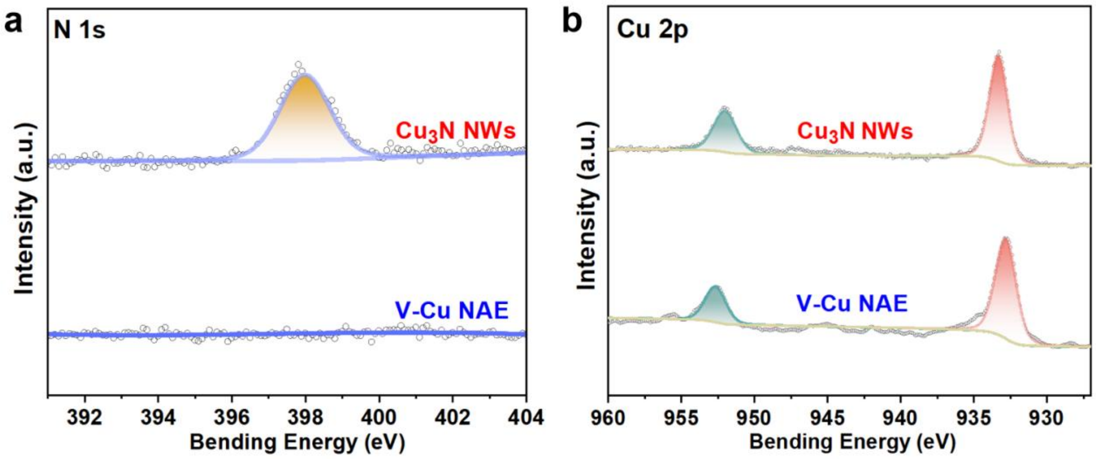

Supplementary Figure 3. **XPS spectra for Cu_3N NWs and V-Cu NAE.** Focusing on the detection and analysis of N (a) and Cu (b) elements. The disappearance of the N signal after activation, suggesting the in-situ reduction of Cu_3N NWs to V-Cu NAE.

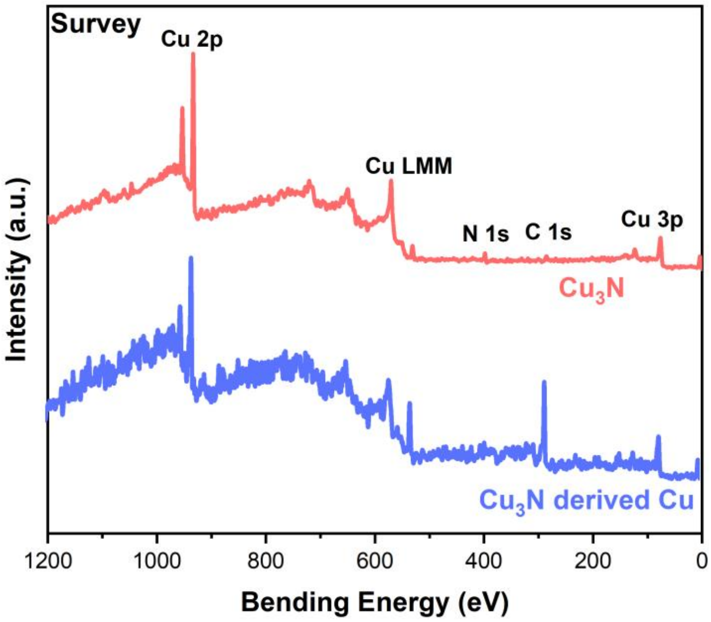

Supplementary Figure 4. **Total (survey) XPS spectra of Cu_3N NWs and V-Cu NAE.**

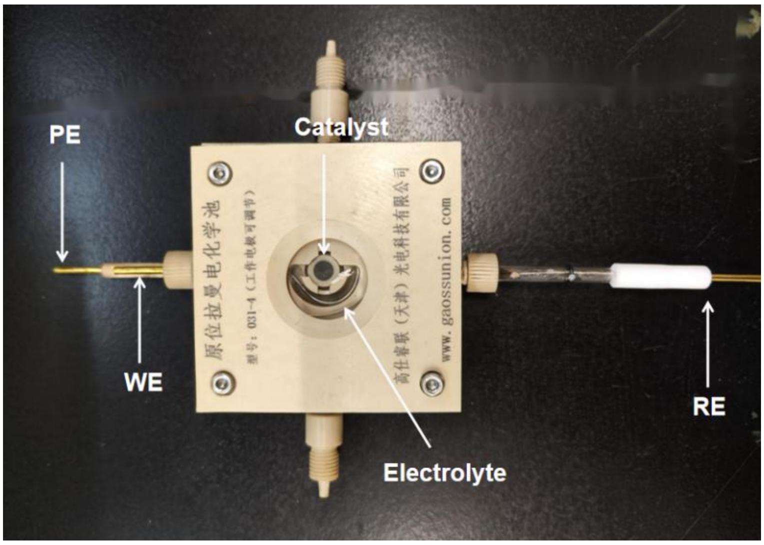

Supplementary Figure 5. **The structure of the in-situ Raman electrolytic cell.** The electrolyte is 0.5 M potassium sulfate, the reference electrode is a saturated calomel electrode, the catalyst is located at the center of the electrolytic cell, in addition to a working electrode and a counter electrode.

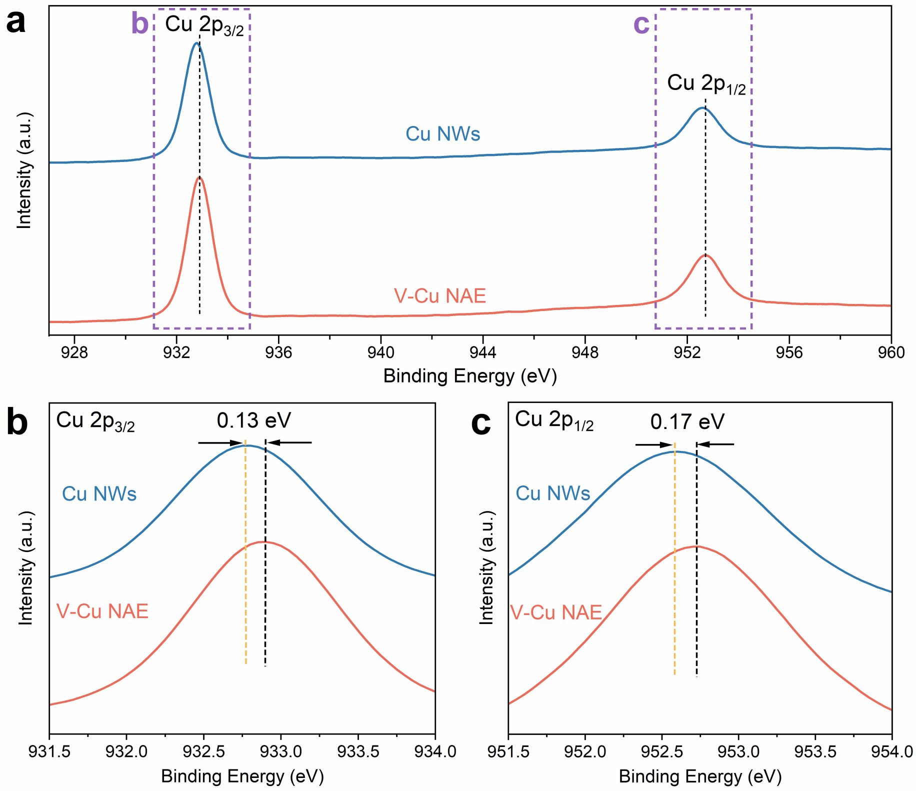

Supplementary Figure 6. (a) XPS spectra of Cu NWs and V-Cu NAE, along with their magnified views in Cu $2p_{3/2}$ (b) and Cu $2p_{1/2}$ (c).

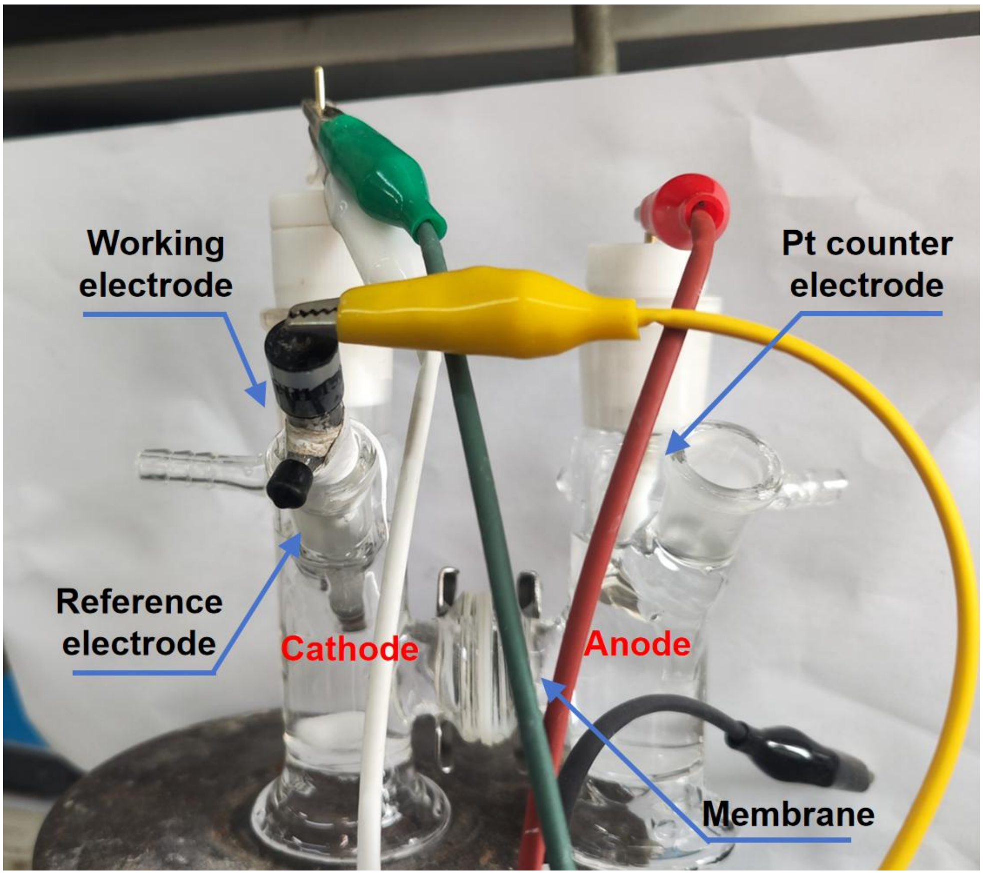

Supplementary Figure 7. Photograph of the H-cell setup used for nitrate reduction experiments, featuring the working, counter, and reference electrodes, along with the ion exchange membrane that separates the electrolyte compartments.

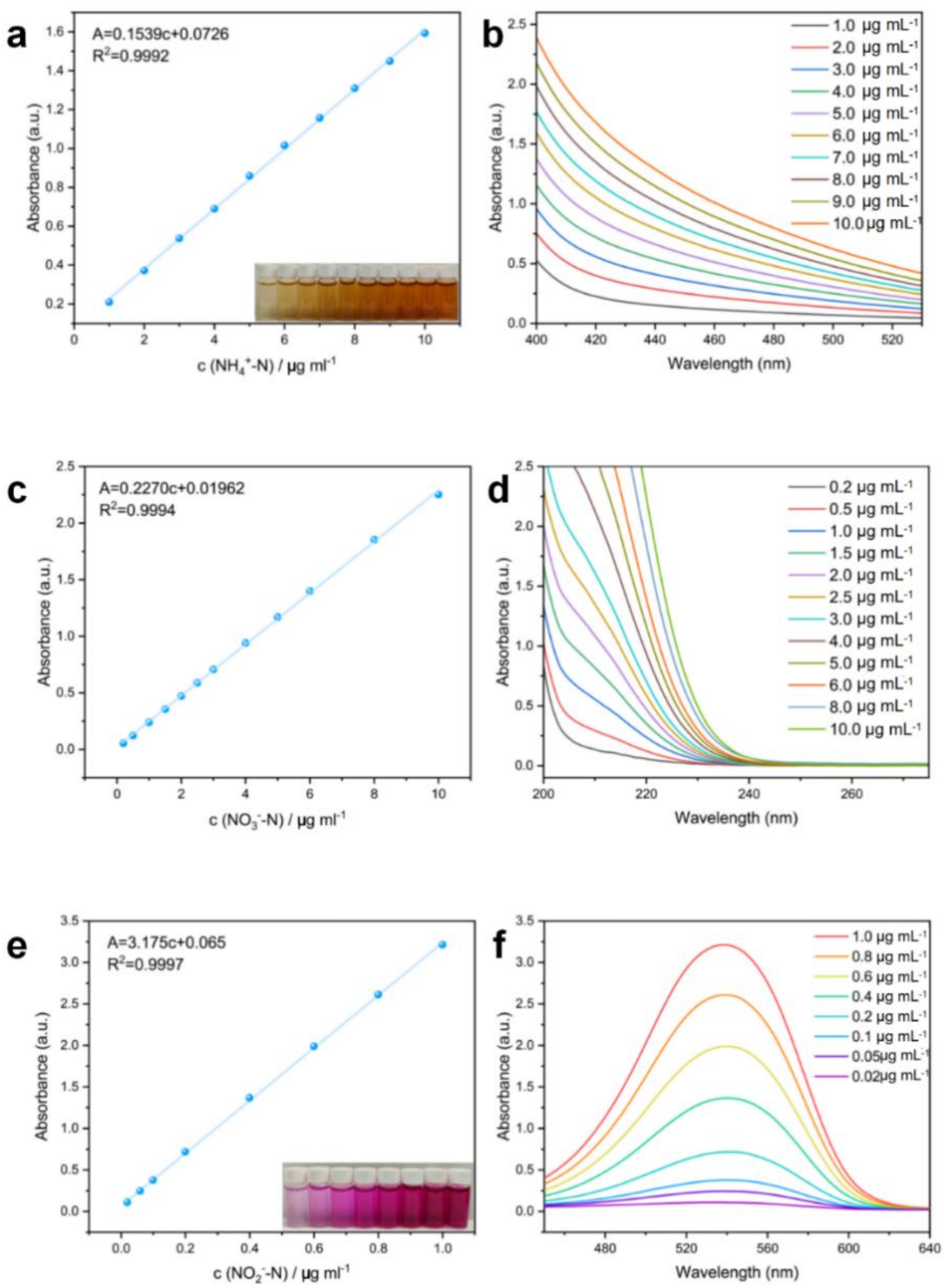

Supplementary Figure 8. **The concentration-absorbance UV-vis calibration curve of NH_4^+, NO_3^- and NO_2^-.** (a), (c) and (e) are the concentration-absorbance calibration curve for NH_4^+, NO_3^-, NO_2^-. (b), (d) and (f) are the corresponding UV absorption spectra for NH_4^+, NO_3^-, NO_2^-.

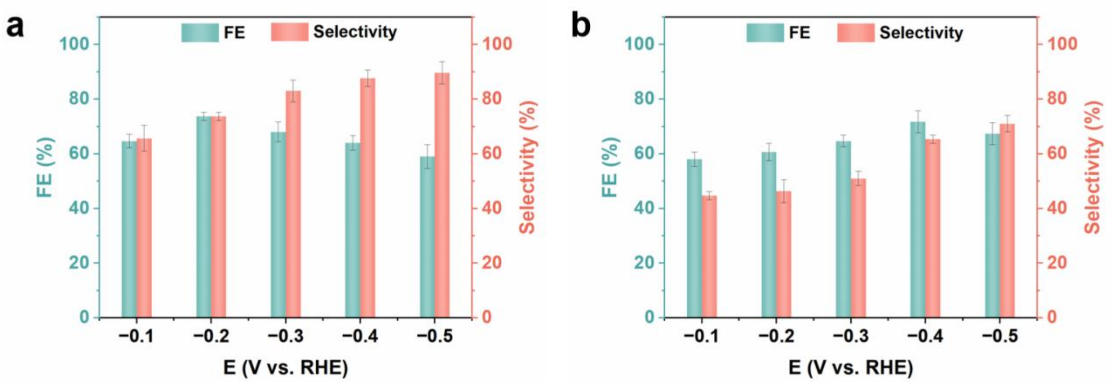

Supplementary Figure 9. Potential-dependent Faradaic efficiency and selectivity of ammonia over (a) Cu_3N NWs and (b) Cu NWs. Error bars represent the standard deviations calculated from three independent measurements.

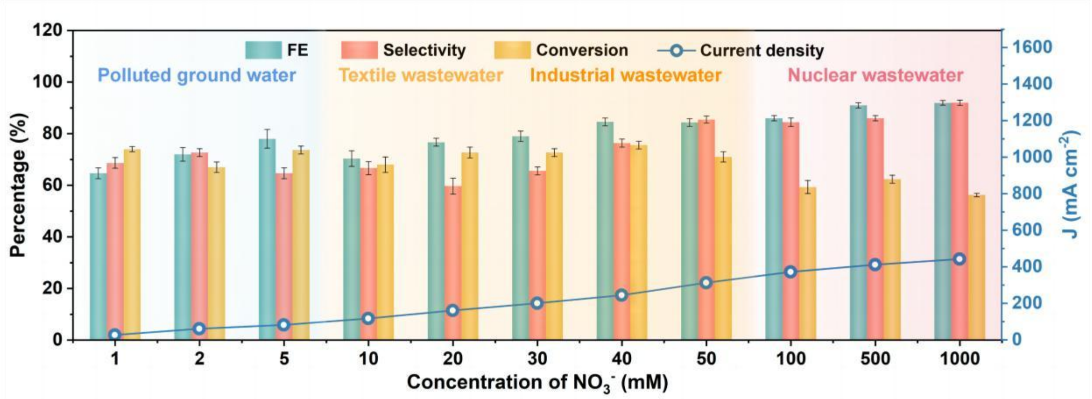

Supplementary Figure 10. Current density, FE of NH_3, selectivity of NH_3, and conversion of NO_3^- in Cu NWs under different concentration nitrate sources. Error bars represent the standard deviations calculated from three independent measurements.

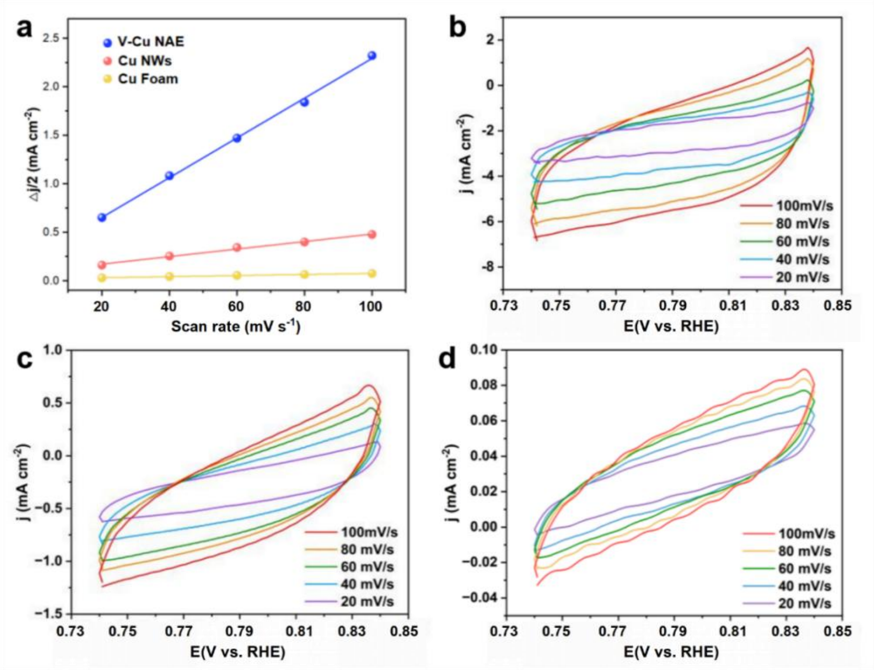

Supplementary Figure 11. **The cyclic voltammograms (CV) scan rate-current relationship and corresponding electrochemical active surface area (ECSA) analysis in 0.5 M K_2SO_4 electrolyte with 200 ppm NO_3^--N.** (a) The electrochemical active surface area (ECSA) analysis for V-Cu NAE, Cu NWs and Cu Foam. The cyclic voltammograms (CV) scan rate-current relationship of (b) V-Cu NAE, (c) Cu NWs and (d) Cu Foam.

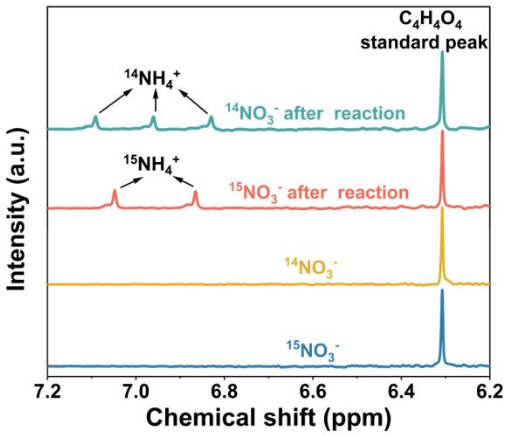

Supplementary Figure 12. **^{1}H NMR spectra before and after NO_3RR using 14.3 mM $^{15}NO_3^-$ and $^{14}NO_3^-$ electrolytes at -0.3 V (vs. RHE).** After electrolysis, distinct peaks corresponding to $^{15}NH_4^+$ and $^{14}NH_4^+$ are observed, whereas these signals are absent in the pre-electrolysis electrolyte.

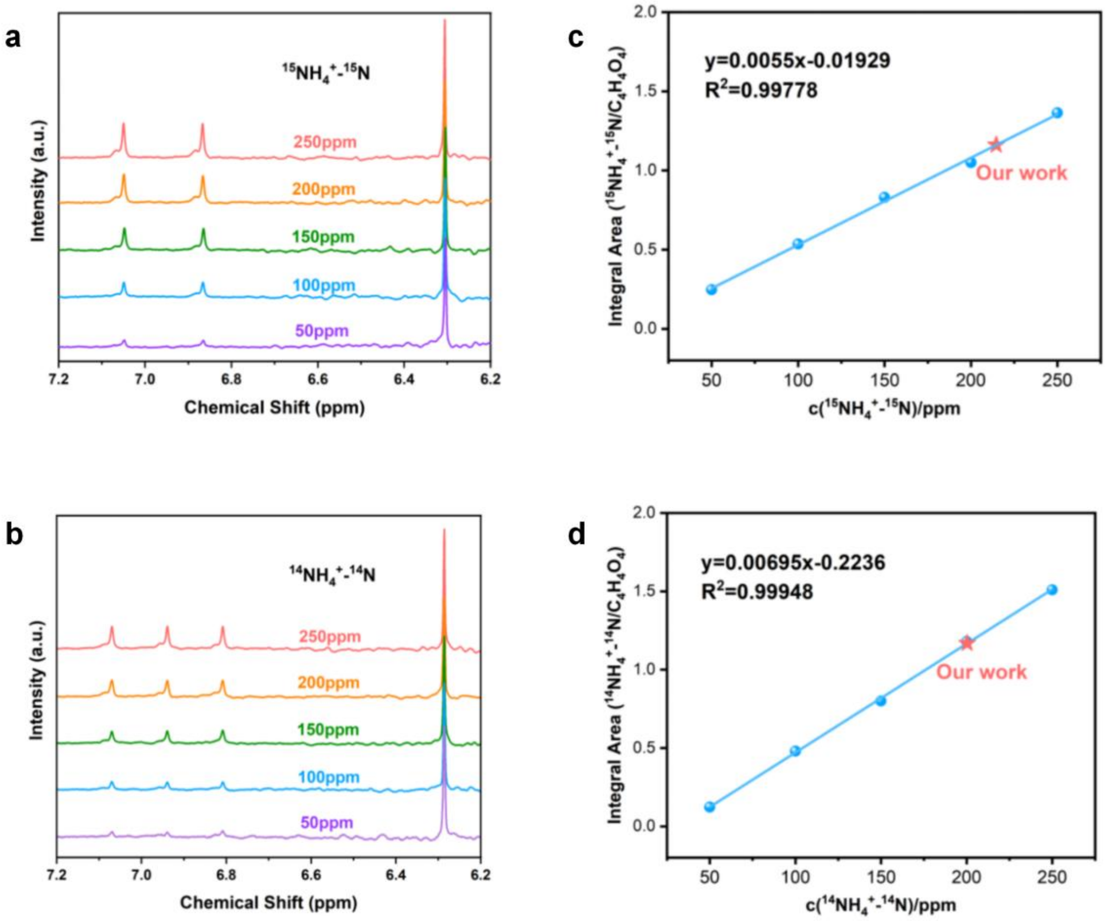

Supplementary Figure 13. The ^{1}H NMR spectra of (a) $^{15}NH_4^+$-^{15}N and (c) $^{14}NH_4^+$-^{14}N were acquired at various concentrations using a 400 MHz spectrometer. The ammonium concentration (NH_4^+-N) was determined quantitatively by ^{1}H NMR using external standards of maleic acid, as the peak area of nuclear magnetic resonance is directly proportional to the ammonium content. The proton signal of maleic acid appeared at a chemical shift of δ = 6.31 ppm. The ^{1}H NMR spectra of $^{15}NH_4^+$ exhibited double peaks at δ = 7.10 and 6.98 ppm, while those of $^{14}NH_4^+$ showed triple peaks at δ = 7.13, 7.05, and 6.96 ppm. (b) A standard curve was constructed by plotting the integral area of $^{15}NH4^+$-^{15}N relative to $C_4H_4O_4$ against the $^{15}NH4^+$-^{15}N concentration. (d) A standard curve was generated by plotting the integral area of $^{14}NH4^+$-^{14}N relative to $C_4H_4O_4$ against the $^{14}NH4^+$-^{14}N concentration.

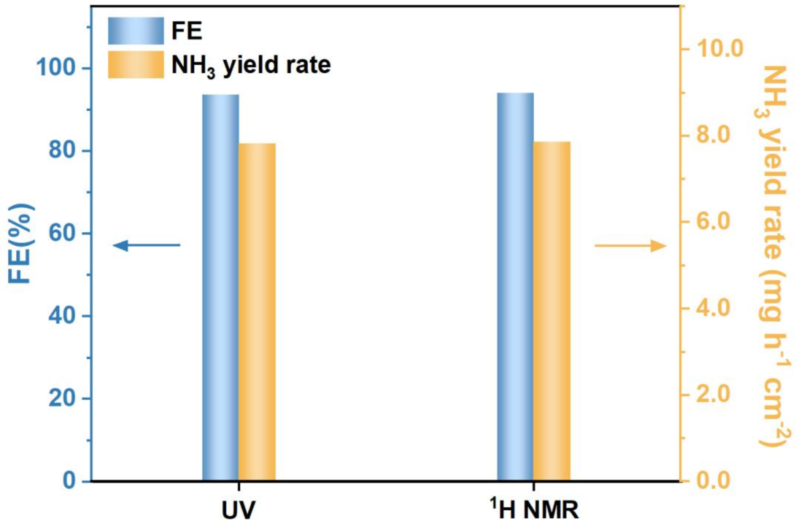

Supplementary Figure 14. Comparison of ammonia yield and Faradaic efficiency obtained by colorimetric method and nuclear magnetic quantification method.

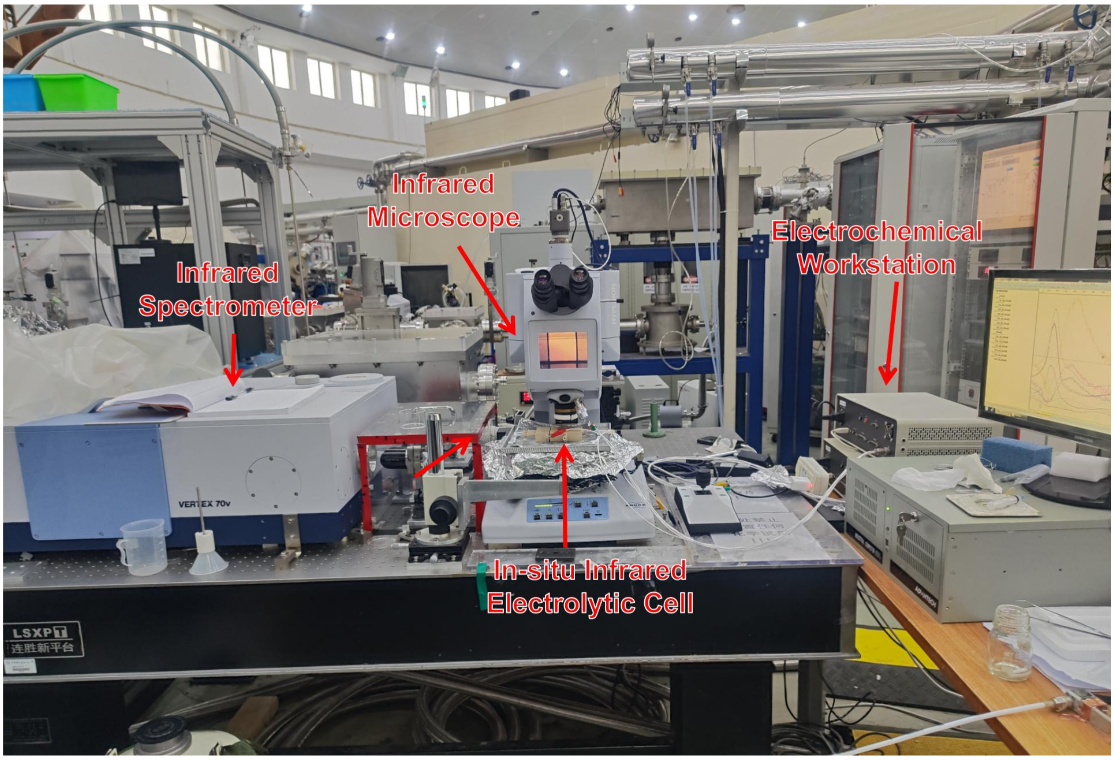

Supplementary Figure 15. Laboratory setup of the synchrotron radiation in-situ infrared apparatus, including an infrared spectrometer, infrared microscope, in-situ infrared electrolytic cell, and electrochemical workstation.

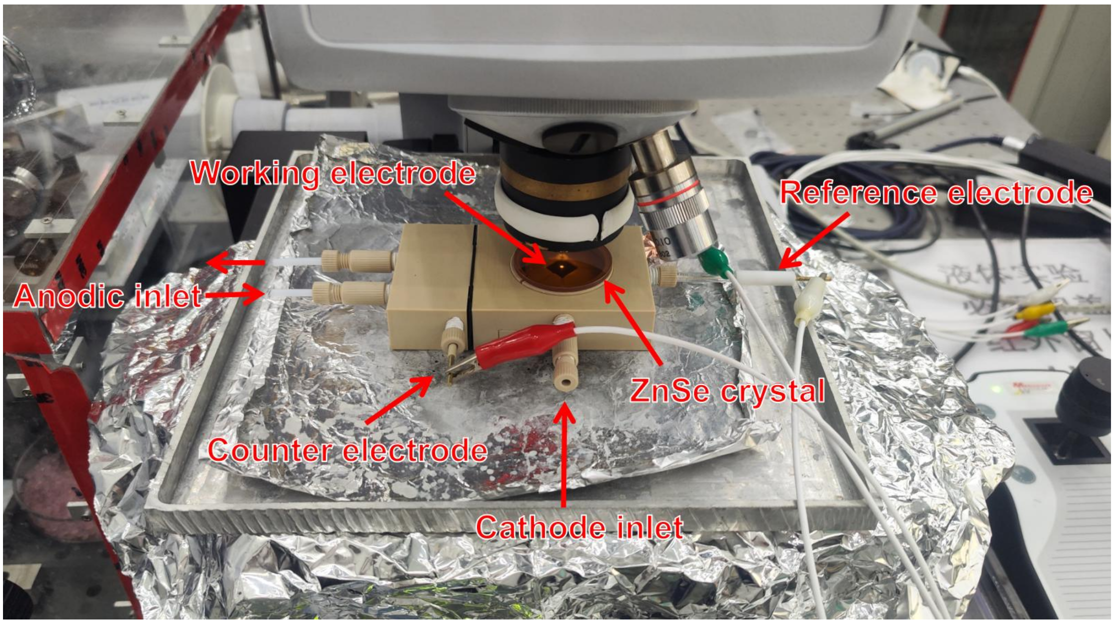

Supplementary Figure 16. In-situ infrared electrolytic cell showing the working electrode, reference electrode, counter electrode, ZnSe crystal, and the electrolyte inlets for the anode and cathode.

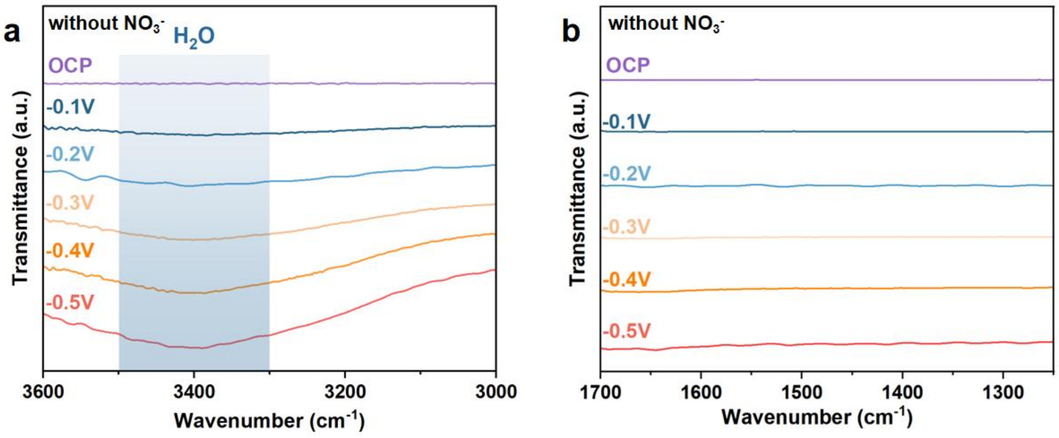

Supplementary Figure 17. **Operando Synchrotron Radiation-FTIR spectroscopy measurements under various potentials for V-Cu NAE during Electrolysis without NO_3^-.** (a) Infrared signals in the range of 3600-3000 cm^{-1}. (b) Infrared signals in the range of 1700-1250 cm^{-1}.

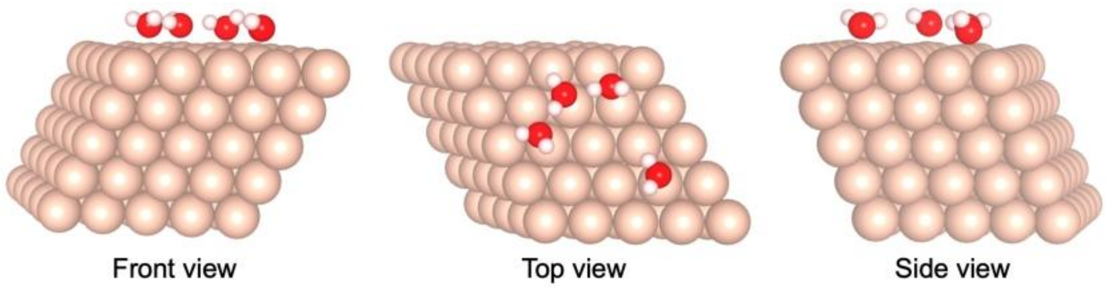

Supplementary Figure 18. Front view, top view, and side view of the Cu (111) surface with four explicit water molecules.

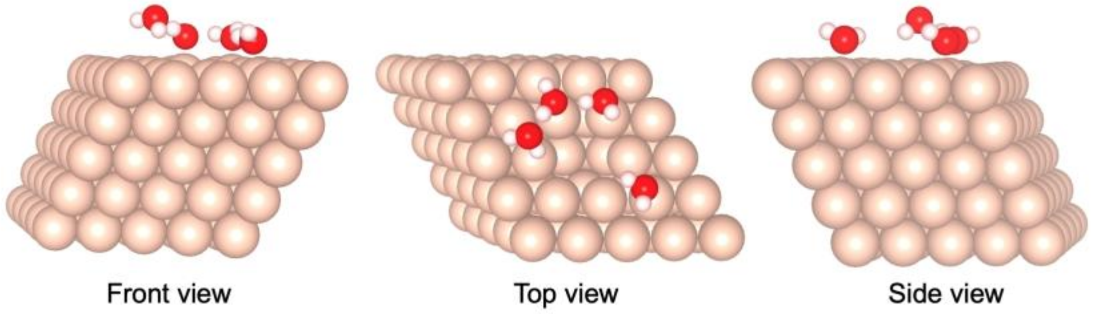

Supplementary Figure 19. Front view, top view, and side view of the V-Cu (111) surface with four explicit water molecules.

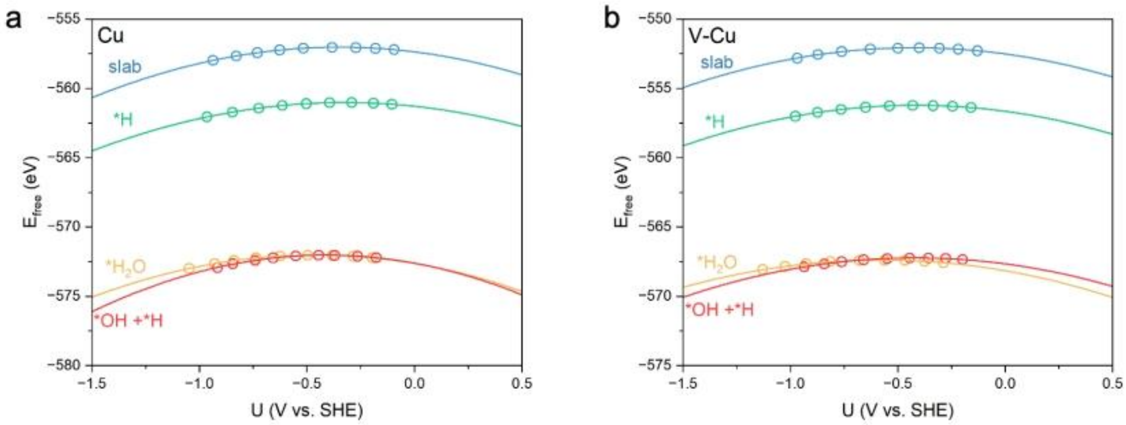

Supplementary Figure 20. Constant potential simulation of water splitting and HER. The $E_{free}(U_{SHE})$ of $*H_2O$, *OH+*H systems for (a) Cu and (b) V-Cu.

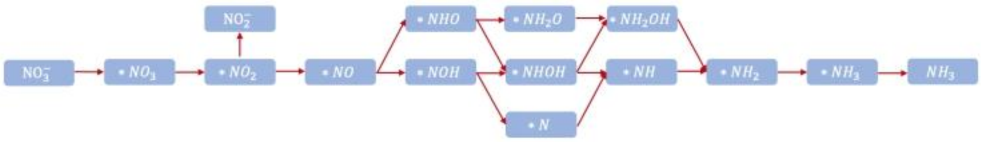

Supplementary Figure 21. **The reaction mechanism of NO3RR and the intermediates involved.** The desorption of nitrite and ammonia and protonation starting from NO are considered.

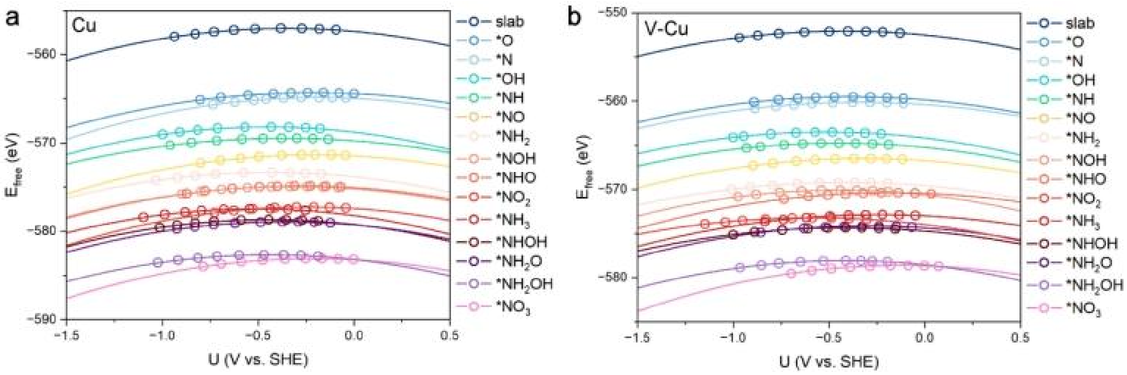

Supplementary Figure 22. The $E_{free}(U_{SHE})$ of slab, *O, *N, *OH, *NH, *NO, *NH$_2$, *NOH, *NHO, *NO$_2$, *NH$_3$, *NHOH, *NH$_2$O, *NH$_2$OH and *NO$_3$ systems for (a) Cu and (b) V-Cu.

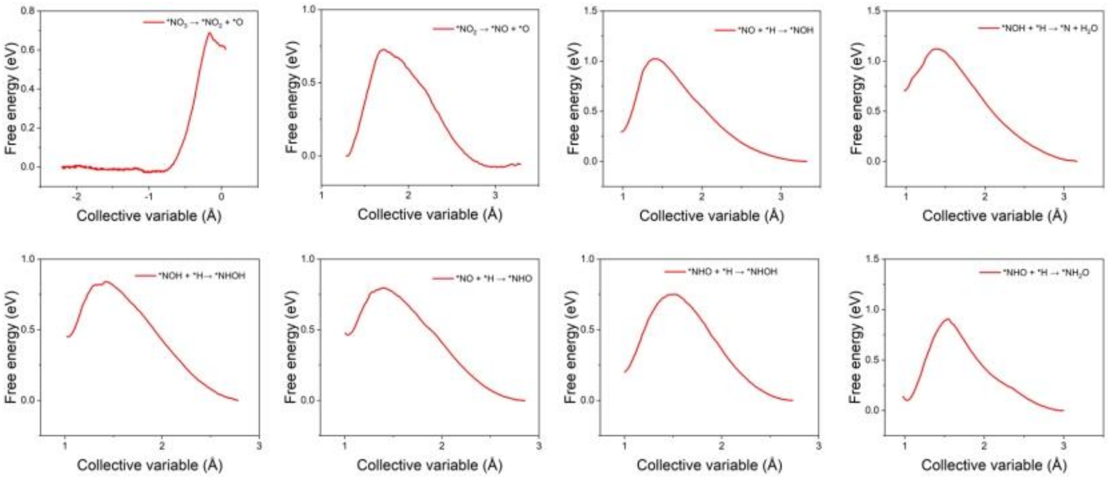

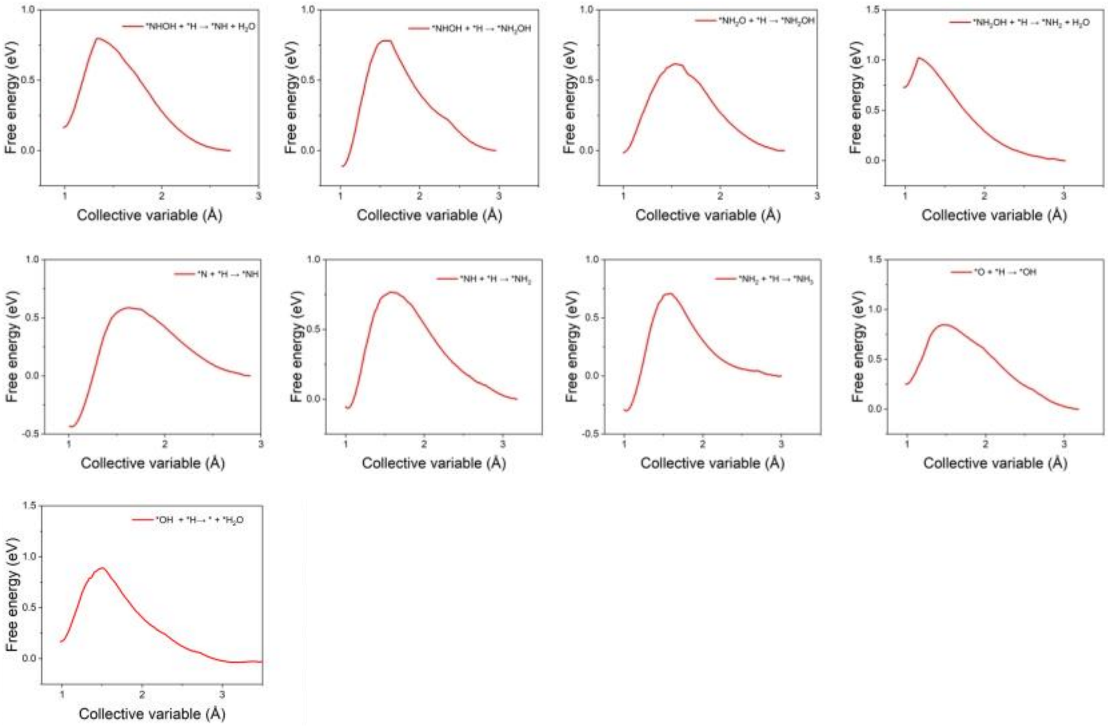

Supplementary Figure 23. Free energy as a function of the CV of NO_3RR pathway by Slow-growth method for Cu.

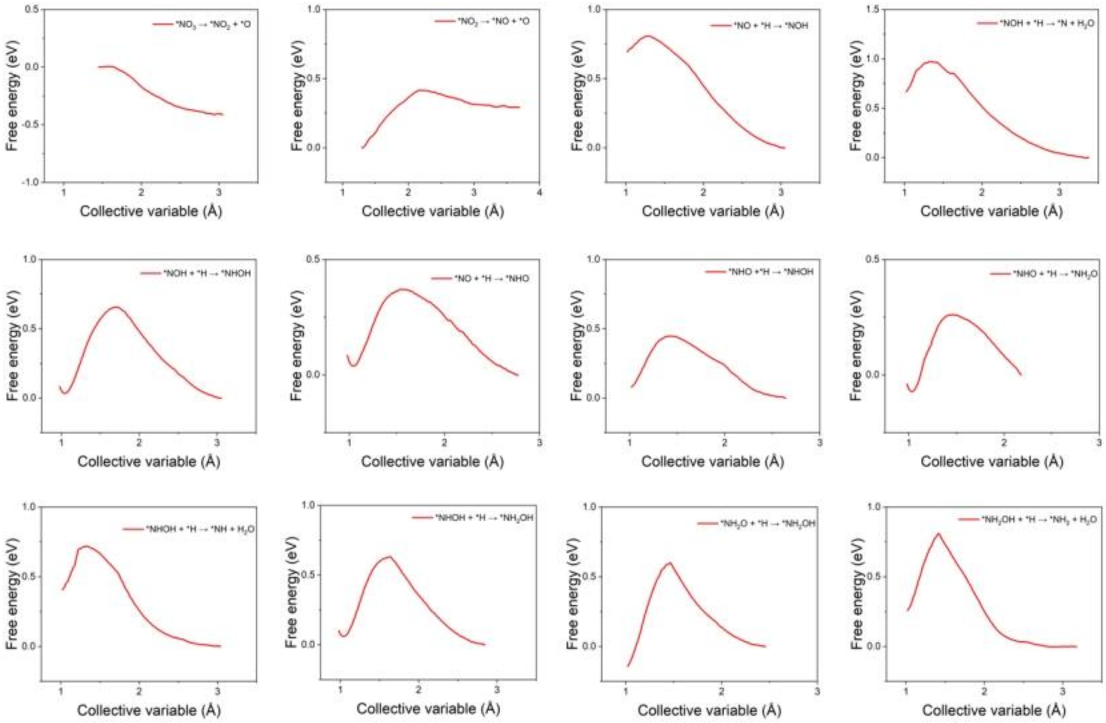

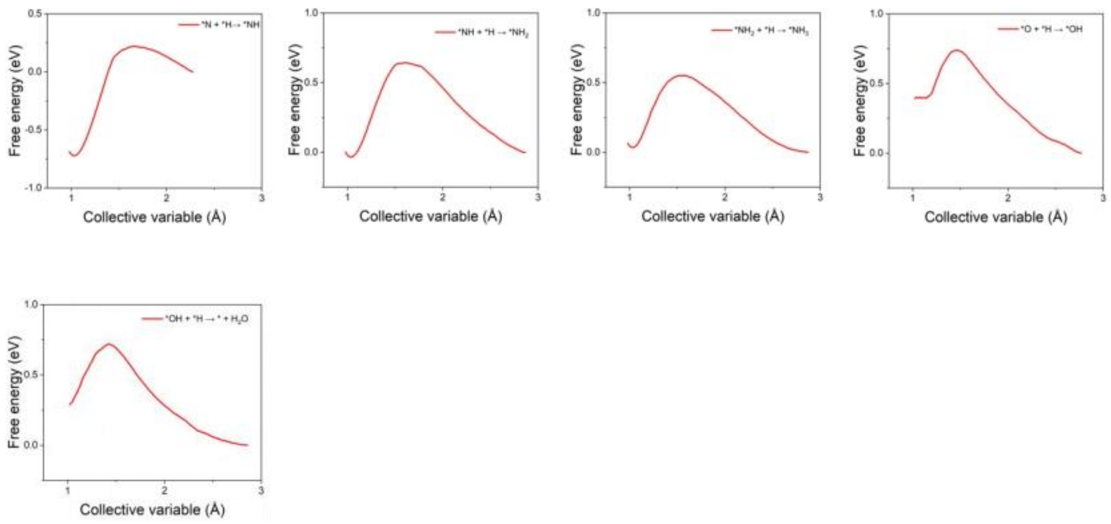

Supplementary Figure 24. Free energy as a function of the CV of NO3RR pathway by Slow-growth method for V-Cu.

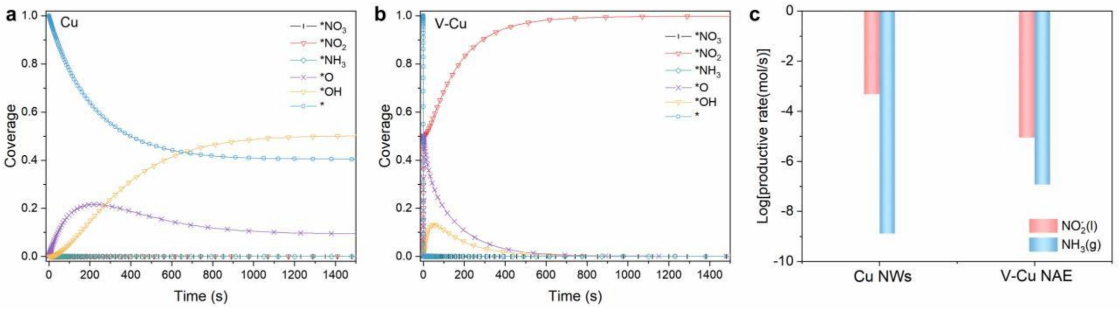

Supplementary Figure 25. The coverage of key intermediates during the reaction, namely *, *NO_3, *NO_2, *O and *OH, as a function of time for (a) Cu and (b) V-Cu. (c) The reaction reaches the steady state, nitrite and ammonia productive rate for Cu and V-Cu.

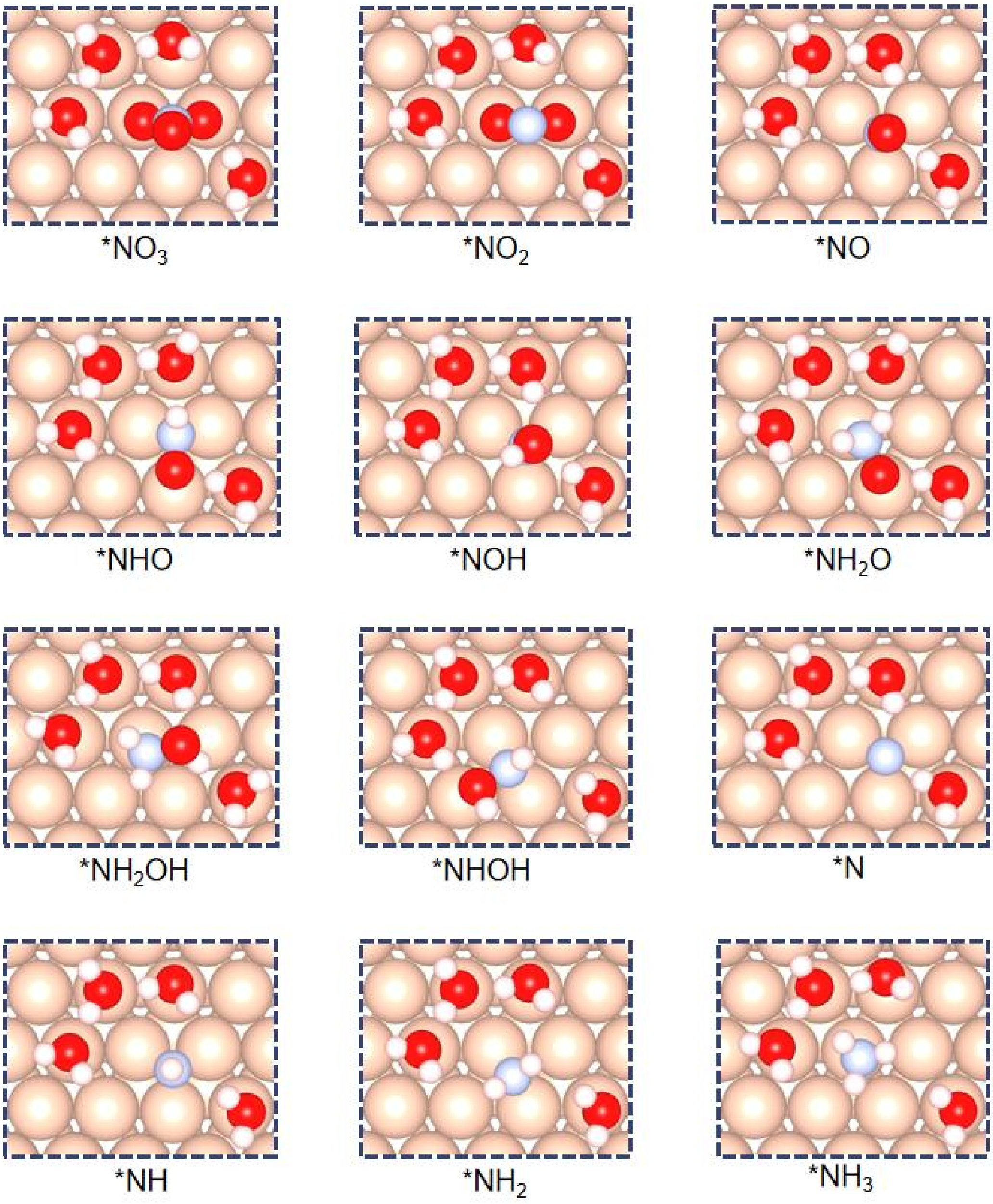

Supplementary Figure 26. The intermediate adsorption structural model for NO_3RR over front view of Cu(111). Orange ball: Cu atom, blue ball: N atom, red ball: O atom, white ball: H atom.

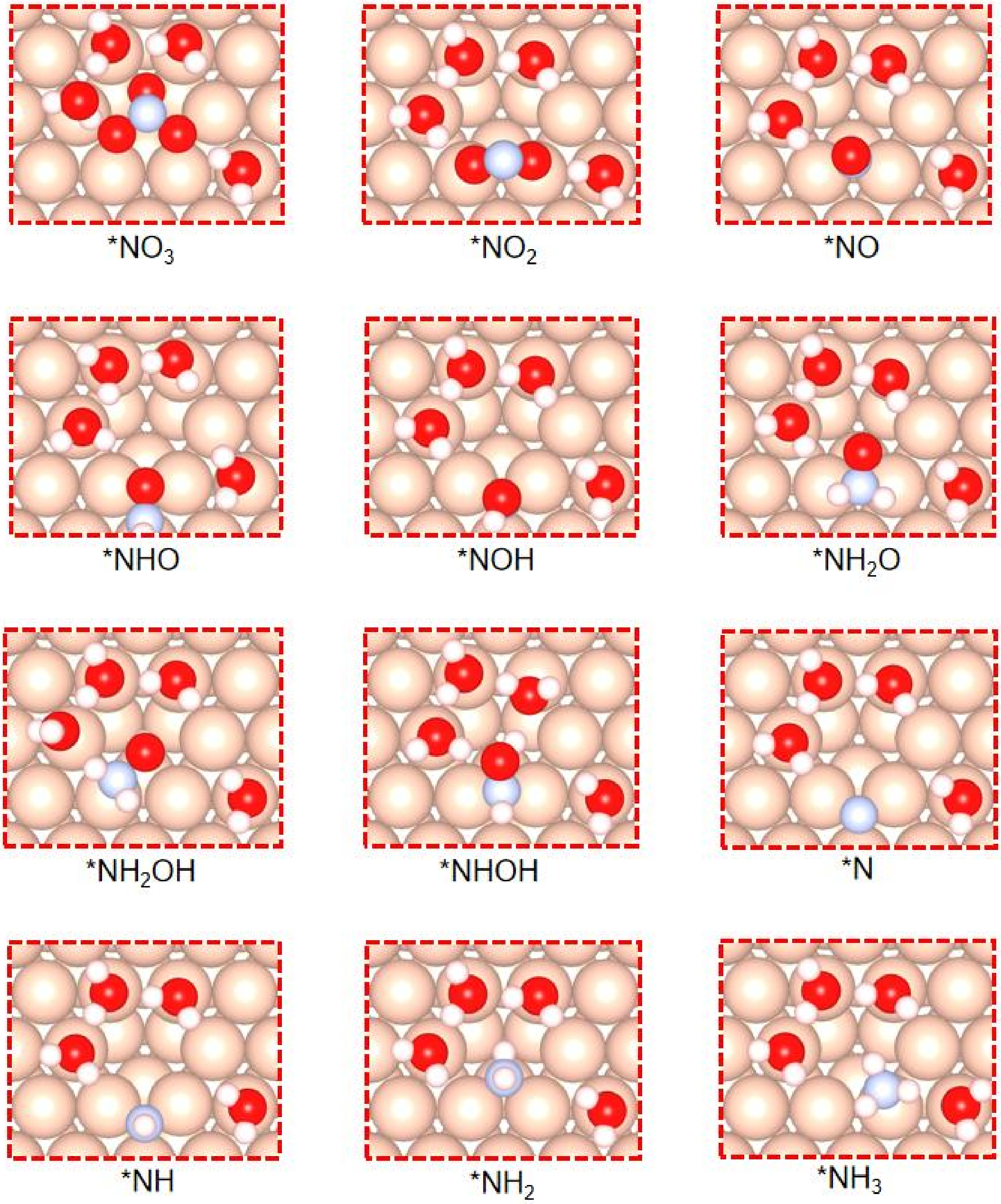

Supplementary Figure 27. **The intermediate adsorption structural model for NO_3RR over front view of V-Cu(111).** Orange ball: Cu atom, blue ball: N atom, red ball: O atom, white ball: H atom.

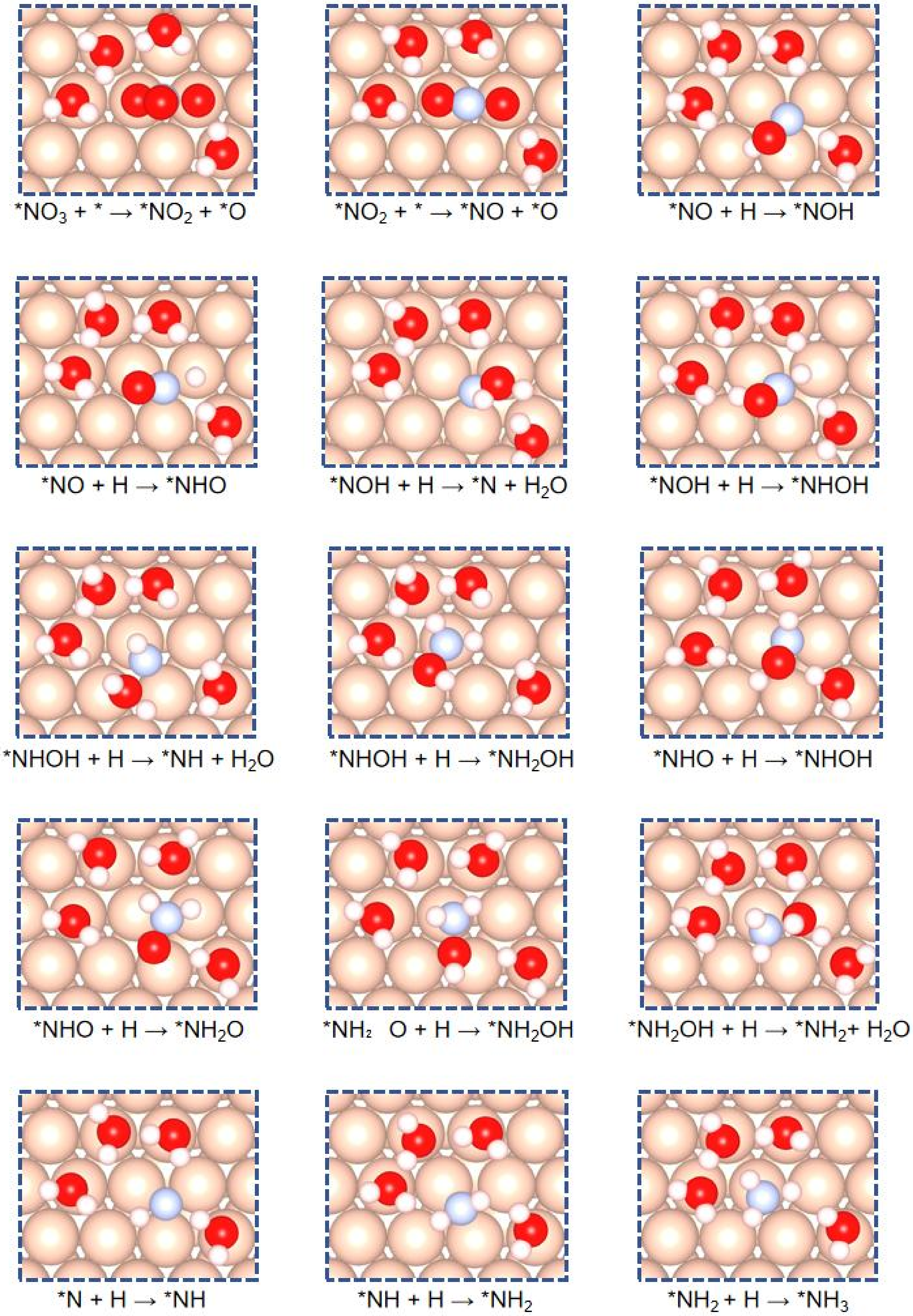

Supplementary Figure 28. **The transition state (TS) structural model for NO_3RR over front view of Cu(111).** Orange ball: Cu atom, blue ball: N atom, red ball: O atom, white ball: H atom.

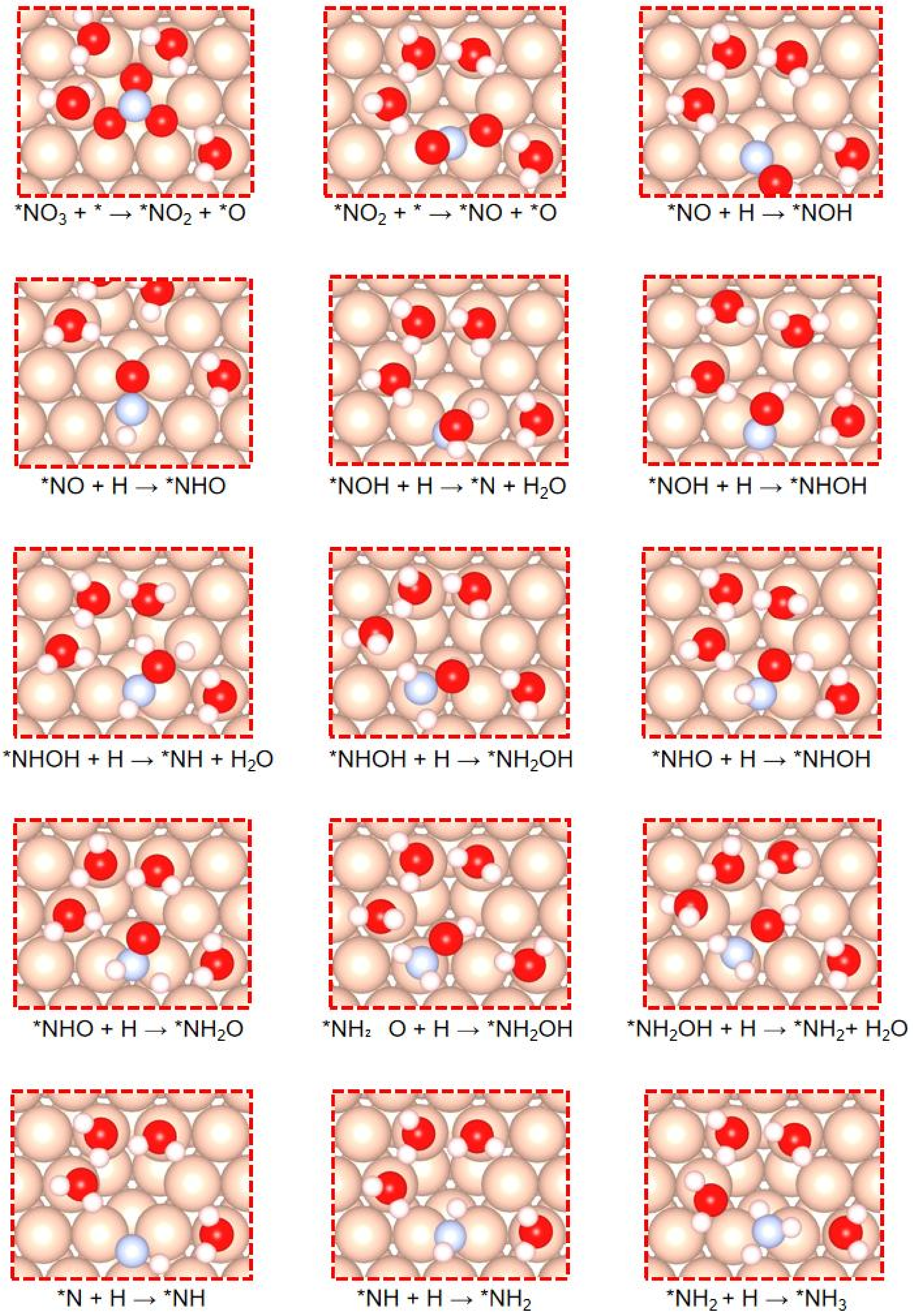

Supplementary Figure 29. **The transition state (TS) structural model for NO_3RR over front view of V-Cu(111).** Orange ball: Cu atom, blue ball: N atom, red ball: O atom, white ball: H atom.

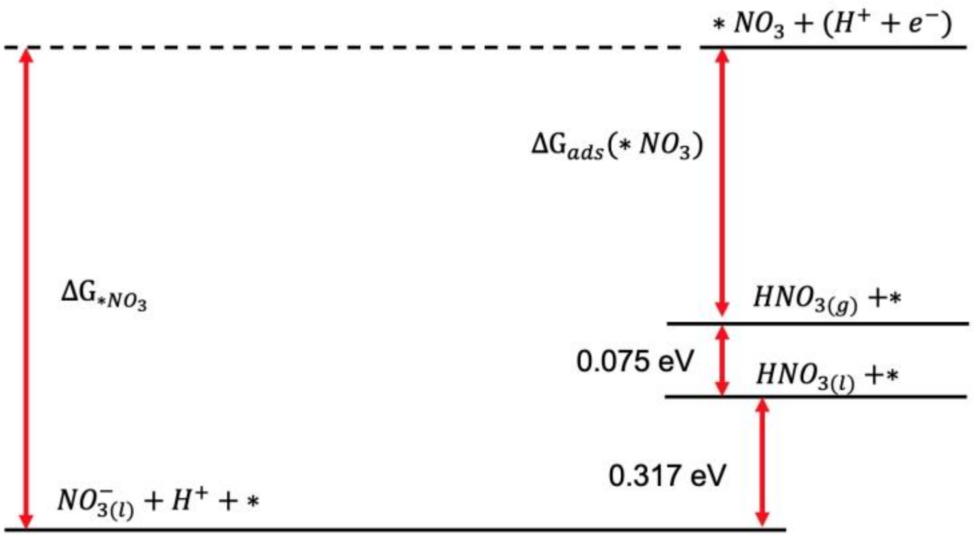

Supplementary Figure 30. The thermodynamic cycle used to calculate the adsorption Gibbs free energy of NO_3^- in the gas phase. The thermodynamic values indicated are obtained from the CRC handbook of chemistry and physics[1].

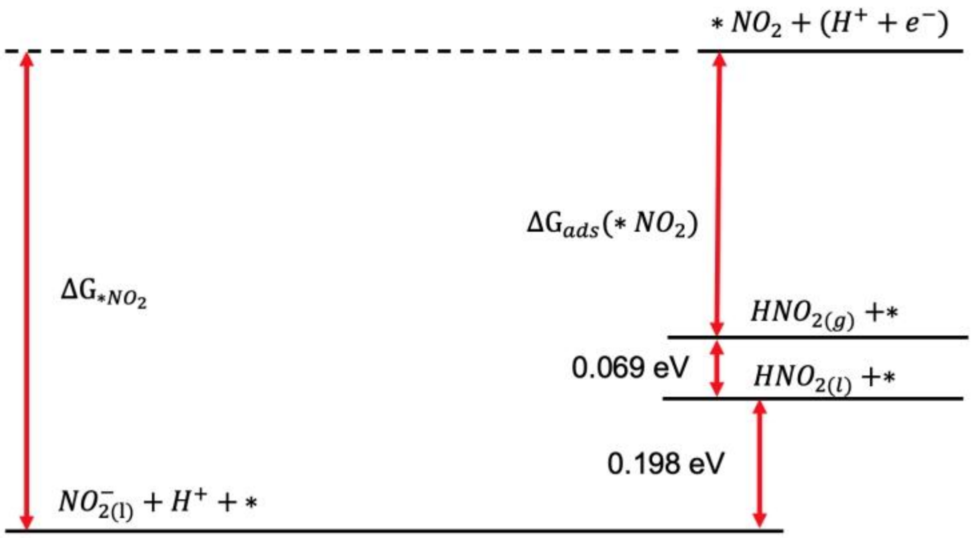

Supplementary Figure 31. The thermodynamic cycle used to calculate the adsorption Gibbs free energy of NO_2^- in the gas phase. The thermodynamic values indicated are obtained from the CRC handbook of chemistry and physics[1].

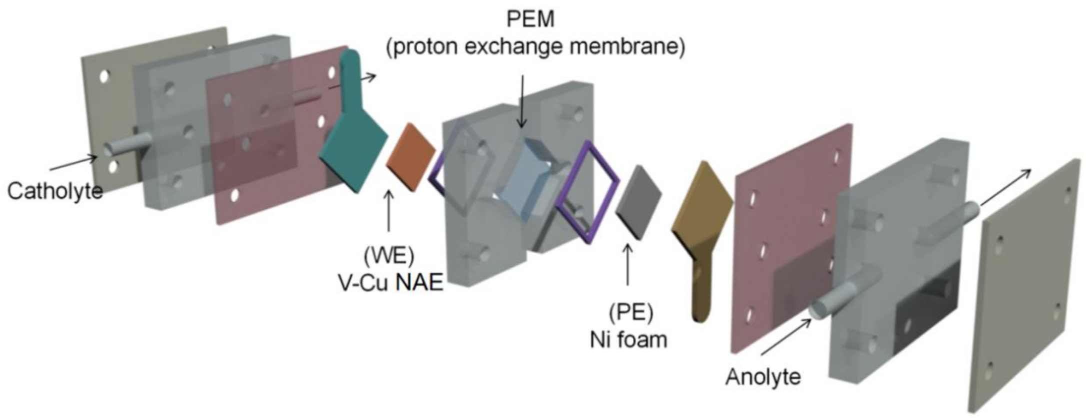

Supplementary Figure 32. **Specific schematic diagram of the two-electrode flow cell device.** The device primarily consists of channels for catholyte and anolyte flow, along with an anode and a cathode, which are separated by a proton exchange membrane. During the testing process, the flow rate of the liquid in the flow cell was maintained at 50 mL/min.

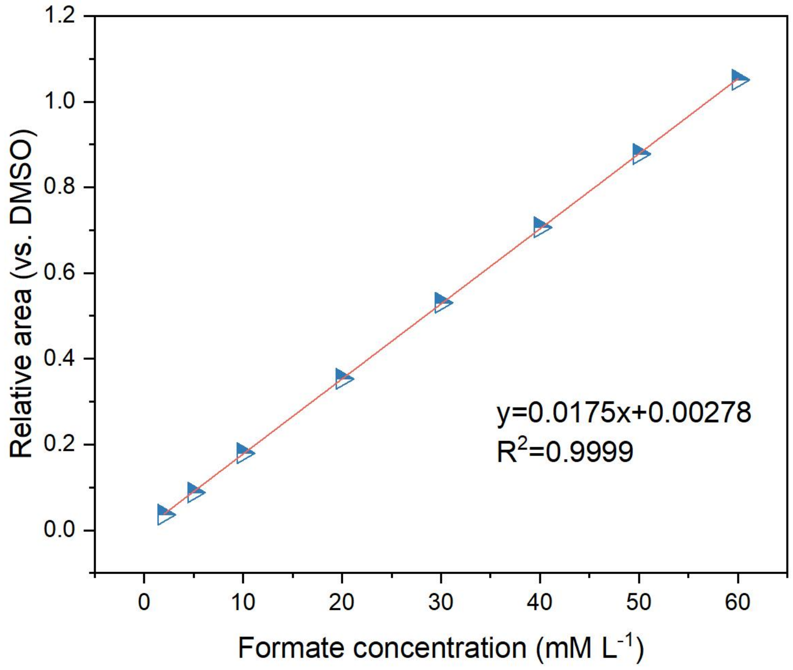

Supplementary Figure 33. A standard curve was generated by plotting the integral area of $HCOO^-$-^1H relative to a specific peak of DMSO against the ^1H concentration in formate ion.

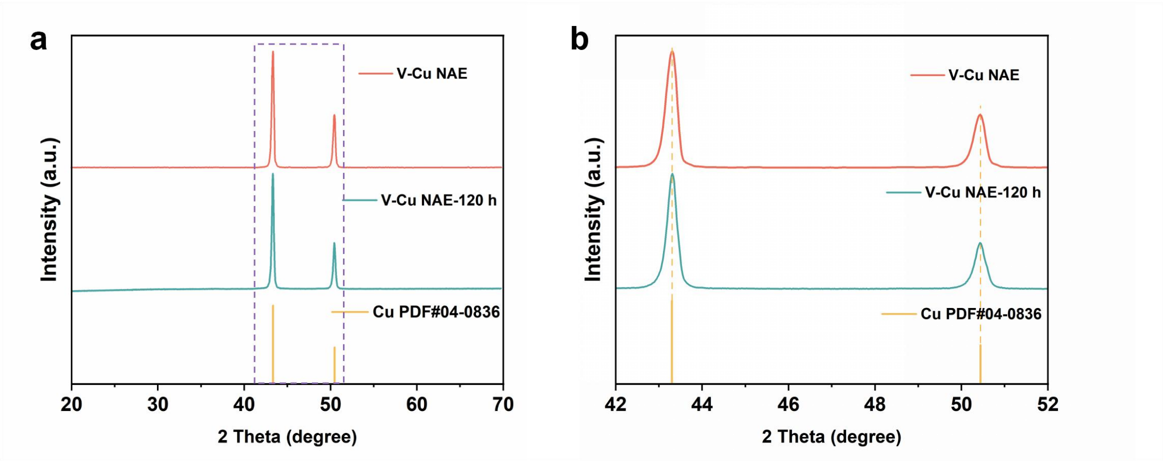

Supplementary Figure 34. (a) XRD spectra of V-Cu NAE catalyst before and after 120 h electrocatalytic NO_3RR. (b) Enlarged XRD spectra of the boxed region from (a).

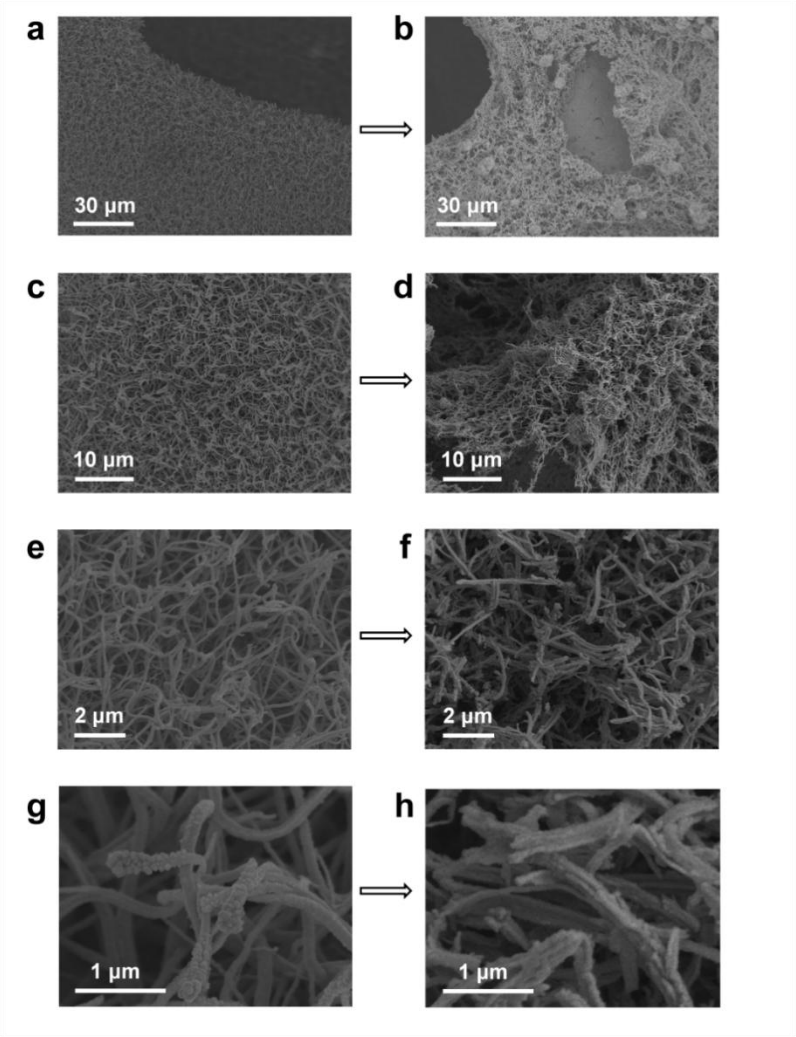

Supplementary Figure 35. SEM images of the V-Cu NAE catalyst before (**a, c, e, g**) and after 120 h (**b, d, f, h**) electrocatalytic NO_3^-RR.

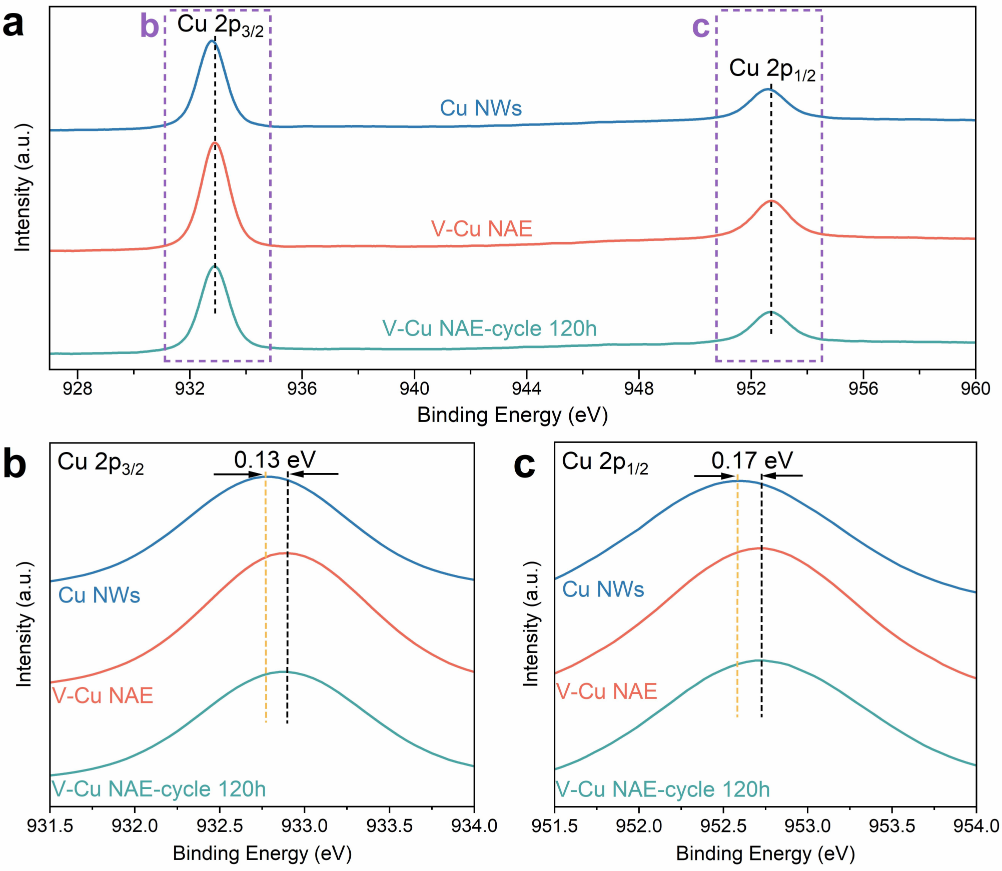

Supplementary Figure 36. (a) XPS spectra of Cu NWs, V-Cu NAE and V-Cu NAE after 120 hours of cycling, along with their magnified views in Cu 2$p_{3/2}$ (b) and Cu 2$p_{1/2}$ (c).

Supplementary Tables

Supplementary Table 1. Comparison of the electrocatalytic activities of V-Cu NAE with other reported materials for NO_3RR.

Material	Electrode	cNO_3^- (mM)	Faradaic efficiency (%)	NH_3 yield rate (mg h^{-1} cm^{-2})	References
Defect-rich Cu NAE	0.5 M K_2SO_4	1	90.6	1.5	This work
		2	90.3	6.1	
		5	92	10.6	
		10	93.7	14.3	
		20	94.8	24.3	
		50	95.8	64.1	
		100	96.1	83.1	
$Ni_3Fe–CO_3$ LDH/Cu foam	1.0 M KOH	1	40.1	0.2	[5]
		2	62.5	0.4	
		5	95.8	1.4	
		10	93.0	2.2	
CuCoSP/ Cu foil	1.0 M KOH	1	90.1	0.2	[6]
		2	92.2	0.6	
		5	92.3	1.3	
		10	92.1	2.6	
		20	93.5	5.6	
		50	94.7	11.7	
		100	89.5	28.9	
$Cu_{50}Ni_{50}$/ Cu-PTFE	1.0 M KOH	1	65.0	0.006	[7]
		2	74.0	0.1	
		10	93.0	0.5	
		50	94.0	2.23	
		100	99.0	4.1	
Rh@Cu NWs	0.1 M Na_2SO_4	2	66.0	1.0	[8]
		10	85.0	3.3	
		50	86.0	5.3	
		100	93.0	15.7	
CoO_x Nanosheets/ carbon cloth	0.1 M KOH	1	43.0	0.5	[9]
		5	49.0	1.7	
		10	83.0	3.1	
		50	78.0	17.1	
		100	92.0	37.3	
Co-doped Fe/Fe_2O_3	0.5 M K_2SO_4	5	42.0	0.16	[10]
		20	64.0	0.6	
		50	87.0	0.9	
		100	66.0	0.92	

Supplementary Table 2. Calculated Gibbs free energies of NO_3RR at -0.289 V vs RHE and T = 298.15 K for Cu and V-Cu.

System	V-Cu	Cu
	Free energy (eV)	Free energy (eV)
$*NO_3$	-1.62	-0.33
$*NO_2$	-3.09	-2.46
*NO	-4.70	-4.82
*NOH	-4.50	-4.38
*N	-6.26	-6.59
*NHOH	-5.37	-4.48
$*NH_2OH$	-5.62	-4.83
$*NH_2O$	-5.46	-4.81
*NHO	-5.70	-4.56
*NH	-7.17	-6.76
$*NH_2$	-8.20	-7.08
$*NH_3$	-8.35	-7.59
NO_2^-	-1.75	-1.75
NH_3	-7.78	-7.78
$*H_2O$	-0.517	-0.286
*H+*OH	-0.824	-0.734
*H	-0.840	-0.636

Supplementary Table 3. Activation energies of NO_3RR at -0.289 V vs RHE and T = 298.15 K for Cu and V-Cu.

Reaction	V-Cu	Cu
	E_a (eV)	E_a (eV)
$*NO_3 + * \rightarrow *NO_2 + *O$	0.010	0.689
$*NO_2 + * \rightarrow *NO + *O$	0.925	0.727
$*NO + H \rightarrow *NHO$	0.618	0.958
$*NO + H \rightarrow *NOH$	1.344	1.069
$*NOH + H \rightarrow *NHOH$	1.093	0.887
$*NOH + H \rightarrow *N + H_2O$	1.620	1.168
$*NHO + H \rightarrow *NHOH$	0.745	0.797
$*NHO + H \rightarrow *NH_2O$	0.434	0.954
$*NHOH + H \rightarrow *NH + H_2O$	1.198	0.842
$*NHOH + H \rightarrow *NH_2OH$	1.051	0.828
$*NH_2O + H \rightarrow *NH_2OH$	1.000	0.661
$*NH_2OH + H \rightarrow *NH_2 + H_2O$	1.349	1.069
$*N + H \rightarrow *NH$	0.371	0.633
$*NH + H \rightarrow *NH_2$	1.072	0.815
$*NH_2 + H \rightarrow *NH_3$	0.920	0.755
$O + H \rightarrow *OH$	1.232	0.892
$*OH + H \rightarrow H_2O + *$	1.199	0.936
$*H_2O + * \rightarrow *H + *OH$	-0.135	0.434

Supplementary Table 4. Parameters for fitting the relationship between E_{free} and U under constant potential simulation for Cu.

	a	b	c	C (e/V)	U_0 (V/SHE)	E_0 (eV)
$*NO_3$	-2.75	-0.68	-578.65	5.49	-0.12	-578.69
$*NO_2$	-2.26	-1.07	-573	4.51	-0.24	-573.13
*NO	-2.41	-1.54	-566.75	4.81	-0.32	-567
*NOH	-2.64	-2.35	-570.62	5.28	-0.45	-571.14
*N	-2.21	-1.51	-560.41	4.42	-0.34	-560.67
*NHOH	-2.21	-1.81	-574.72	4.43	-0.41	-575.09
$*NH_2OH$	-2.65	-2.22	-578.55	5.29	-0.42	-579.01
$*NH_2O$	-2.44	-1.47	-574.37	4.87	-0.3	-574.59
*NHO	-2.31	-0.82	-570.45	4.62	-0.18	-570.53
*NH	-2.37	-2.14	-565.25	4.74	-0.45	-565.74
$*NH_2$	-2.16	-1.77	-569.59	4.31	-0.41	-569.95
$*NH_3$	-2.24	-2.61	-573.99	4.47	-0.58	-574.75
*H	-2.48	-2.07	-556.66	4.97	-0.42	-557.09
*O	-2.34	-1.81	-559.88	4.67	-0.39	-560.23
*OH	-2.52	-2.59	-564.19	5.04	-0.51	-564.86
slab	-2.47	-2.09	-552.53	4.93	-0.42	-552.97
$*H_2O$	-2.32	-2.68	-568.17	4.64	-0.58	-568.95
*H + *OH	-2.43	-2.04	-567.67	4.85	-0.42	-568.10

Supplementary Table 5. Parameters for fitting the relationship between E_{free} and U under constant potential simulation for V-Cu.

	a	b	c	C (e/V)	U_0 (V/SHE)	E_0 (eV)
*NO_3	-2.7	-1.14	-583.23	5.4	-0.21	-583.35
*NO_2	-2.72	-1.31	-577.44	5.44	-0.24	-577.6
*NO	-2.63	-1.09	-571.48	5.26	-0.21	-571.6
*NOH	-2.44	-1.54	-557.38	4.89	-0.31	-557.62
*N	-2.74	-1.03	-564.98	5.48	-0.19	-565.08
*NHOH	-2.73	-2.42	-574.72	5.46	-0.44	-575.26
*NH_2OH	-2.68	-2.36	-583.21	5.35	-0.44	-583.73
*NH_2O	-2.44	-1.47	-574.37	4.87	-0.3	-574.59
*NHO	-2.43	-1.35	-575.08	4.86	-0.28	-575.27
*NH	-2.22	-1.6	-569.77	4.45	-0.36	-570.06
*NH_2	-2.54	-2.21	-573.84	5.08	-0.44	-574.33
*NH_3	-2.78	-2.86	-578.23	5.55	-0.52	-578.97
*H	-2.53	-1.65	-561.30	5.06	-0.33	-561.57
*O	-2.34	-1.81	-559.88	4.67	-0.39	-560.23
*OH	-2.82	-2.55	-568.79	5.64	-0.45	-569.37
slab	-2.75	-1.92	-552.53	5.5	-0.35	-552.86
*H_2O	-2.83	-2.61	-572.64	5.64	-0.46	-573.24
*H + *OH	-3.44	-2.83	-572.62	6.88	-0.41	-573.21

Supplementary Table 6. Parameters used by Slow-growth approach for Cu.

Slow Growth Approach in Reaction Networks	CV (Å)	Transformation velocity (Å·fs^{-1})
$*NO_3 + * \rightarrow *NO_2 + *O$	d(N-O)-d(Cu-O)	0.0003 (t=10000 fs)
$*NO_2 + * \rightarrow *NO + *O$	d(N-O)	0.0002 (t=10000 fs)
$*NO + *H \rightarrow *NOH + *$	d(O-H)	-0.0002345 (t=10000 fs)
$*NO + *H \rightarrow *NHO + *$	d(N-H)	-0.000185 (t=10000 fs)
$*NOH + *H \rightarrow *NHOH + *$	d(N-H)	-0.000176 (t=10000 fs)
$*NOH + *H \rightarrow *N + H_2O + *$	d(O-H)	-0.0002182 (t=10000 fs)
$*NHO + *H \rightarrow *NH_2O + *$	d(N-H)	-0.0002018 (t=10000 fs)
$*NHO + *H \rightarrow *NHOH + *$	d(O-H)	-0.0002676 (t=10000 fs)
$*NHOH + *H \rightarrow *NH_2OH + *$	d(N-H)	-0.0001937 (t=10000 fs)
$*NHOH + *H \rightarrow *NH + H_2O + *$	d(O-H)	-0.000172 (t=10000 fs)
$*NH_2O + *H \rightarrow *NH_2OH + *$	d(O-H)	-0.000166 (t=10000 fs)
$*NH_2OH + *H \rightarrow *NH_2 + H_2O + *$	d(O-H)	-0.0002036 (t=10000 fs)
$*N + *H \rightarrow *NH + *$	d(N-H)	-0.000188 (t=10000 fs)
$*NH + *H \rightarrow *NH_2 + *$	d(N-H)	-0.000218 (t=10000 fs)
$*NH_2 + *H \rightarrow *NH_3 + *$	d(N-H)	-0.0002 (t=10000 fs)
$*O + *H \rightarrow *OH + *$	d(O-H)	-0.00022 (t=10000 fs)
$*OH + *H \rightarrow *H_2O + *$	d(O-H)	-0.0002856 (t=10000 fs)

Supplementary Table 7. Parameters used by Slow-growth approach for V-Cu.

Slow Growth Approach in Reaction Networks	CV (Å)	Transformation velocity ($Å·fs^{-1}$)
$*NO_3 + * \rightarrow *NO_2 + *O$	d(N-O)	0.0003 (t=4000 fs)
$*NO_2 + * \rightarrow *NO + *O$	d(N-O)	0.0003 (t=6000 fs)
$*NO + *H \rightarrow *NOH + *$	d(O-H)	-0.000203 (t=10000 fs)
$*NO + *H \rightarrow *NHO + *$	d(N-H)	-0.0001794 (t=10000 fs)
$*NOH + *H \rightarrow *NHOH + *$	d(N-H)	-0.0002356 (t=10000 fs)
$*NOH + *H \rightarrow *N + H_2O + *$	d(O-H)	-0.0002075 (t=10000 fs)
$*NHO + *H \rightarrow *NH_2O + *$	d(N-H)	-0.0001202 (t=10000 fs)
$*NHO + *H \rightarrow *NHOH + *$	d(O-H)	-0.0001619 (t=10000 fs)
$*NHOH + *H \rightarrow *NH_2OH + *$	d(N-H)	-0.0001866 (t=10000 fs)
$*NHOH + *H \rightarrow *NH + H_2O + *$	d(O-H)	-0.0002017 (t=10000 fs)
$*NH_2O + *H \rightarrow *NH_2OH + *$	d(O-H)	-0.0001438 (t=10000 fs)
$*NH_2OH + *H \rightarrow *NH_2 + H_2O + *$	d(O-H)	-0.0002157 (t=10000 fs)
$*N + *H \rightarrow *NH + *$	d(N-H)	-0.0001297 (t=10000 fs)
$*NH + *H \rightarrow *NH_2 + *$	d(N-H)	-0.0001886 (t=10000 fs)
$*NH_2 + *H \rightarrow *NH_3 + *$	d(N-H)	-0.0001893 (t=10000 fs)
$*O + *H \rightarrow *OH + *$	d(O-H)	-0.000175 (t=10000 fs)
$*OH + *H \rightarrow *H_2O + *$	d(O-H)	-0.0001838 (t=10000 fs)

Supplementary Table 8. Calculated Gibbs free energies of NO_3RR at -0.289 V vs RHE and T = 298.15 K for Cu and V-Cu.

System	V-Cu	Cu
	Free energy (eV)	Free energy (eV)
$*NO_3$	-1.62	-0.33
$*NO_2$	-3.09	-2.46
*NO	-4.70	-4.82
*NOH	-4.50	-4.38
*N	-6.26	-6.59
*NHOH	-5.37	-4.48
$*NH_2OH$	-5.62	-4.83
$*NH_2O$	-5.46	-4.81
*NHO	-5.70	-4.56
*NH	-7.17	-6.76
$*NH_2$	-8.20	-7.08
$*NH_3$	-8.35	-7.59
NO_2^-	-1.75	-1.75
NH_3	-7.78	-7.78
$*H_2O$	-0.517	-0.286
*H+*OH	-0.824	-0.734
*H	-0.840	-0.636

Supplementary Table 9. Activation energies of NO_3RR at -0.289 V vs RHE and T = 298.15 K for Cu and V-Cu.

Reaction	V-Cu	Cu
	E_a (eV)	E_a (eV)
$*NO_3 + * \rightarrow *NO_2 + *O$	0.010	0.689
$*NO_2 + * \rightarrow *NO + *O$	0.925	0.727
$*NO + H \rightarrow *NHO$	0.618	0.958
$*NO + H \rightarrow *NOH$	1.344	1.069
$*NOH + H \rightarrow *NHOH$	1.093	0.887
$*NOH + H \rightarrow *N + H_2O$	1.620	1.168
$*NHO + H \rightarrow *NHOH$	0.745	0.797
$*NHO + H \rightarrow *NH_2O$	0.434	0.954
$*NHOH + H \rightarrow *NH + H_2O$	1.198	0.842
$*NHOH + H \rightarrow *NH_2OH$	1.051	0.828
$*NH_2O + H \rightarrow *NH_2OH$	1.000	0.661
$*NH_2OH + H \rightarrow *NH_2 + H_2O$	1.349	1.069
$*N + H \rightarrow *NH$	0.371	0.633
$*NH + H \rightarrow *NH_2$	1.072	0.815
$*NH_2 + H \rightarrow *NH_3$	0.920	0.755
$O + H \rightarrow *OH$	1.232	0.892
$*OH + H \rightarrow H_2O + *$	1.199	0.936
$*H_2O + * \rightarrow *H + *OH$	-0.135	0.434

Supplementary Notes

Supplementary Note S1. Details of Microkinetic Simulations

The intermediate reactions considered are:

$$NO_3^- + * \underset{k_1^-}{\overset{k_1^+}{\rightleftharpoons}} *NO_3 + e^- \quad (r_1)$$

$$*NO_3 + * \underset{k_2^-}{\overset{k_2^+}{\rightleftharpoons}} *NO_2 + *O \quad (r_2)$$

$$*NO + (H^+ + e^-) \underset{k_3^-}{\overset{k_3^+}{\rightleftharpoons}} *NOH \quad (r_3)$$

$$*NO + (H^+ + e^-) \underset{k_4^-}{\overset{k_4^+}{\rightleftharpoons}} *NHO \quad (r_4)$$

$$*NOH + (H^+ + e^-) \underset{k_5^-}{\overset{k_5^+}{\rightleftharpoons}} *NHOH \quad (r_5)$$

$$*NHO + (H^+ + e^-) \underset{k_6^-}{\overset{k_6^+}{\rightleftharpoons}} *NHOH \quad (r_6)$$

$$*NHO + (H^+ + e^-) \underset{k_7^-}{\overset{k_7^+}{\rightleftharpoons}} *NH_2O \quad (r_7)$$

$$*NHOH + (H^+ + e^-) \underset{k_8^-}{\overset{k_8^+}{\rightleftharpoons}} *NH + H_2O \quad (r_8)$$

$$*NHOH + (H^+ + e^-) \underset{k_9^-}{\overset{k_9^+}{\rightleftharpoons}} *NH_2OH \quad (r_9)$$

$$*NH_2O + (H^+ + e^-) \underset{k_{10}^-}{\overset{k_{10}^+}{\rightleftharpoons}} *NH_2OH \quad (r_{10})$$

$$*NOH + (H^+ + e^-) \underset{k_{11}^-}{\overset{k_{11}^+}{\rightleftharpoons}} *N + H_2O \quad (r_{11})$$

$$*N + (H^+ + e^-) \underset{k_{12}^-}{\overset{k_{12}^+}{\rightleftharpoons}} *NH \quad (r_{12})$$

$$*NH + (H^+ + e^-) \underset{k_{13}^-}{\overset{k_{13}^+}{\rightleftharpoons}} *NH_2 \quad (r_{13})$$

$$* NH_2OH + (H^+ + e^-) \underset{k_{14}^-}{\overset{k_{14}^+}{\rightleftharpoons}} * NH_2 + H_2O \quad (r_{14})$$

$$* NH_2 + (H^+ + e^-) \underset{k_{15}^-}{\overset{k_{15}^+}{\rightleftharpoons}} * NH_3 \quad (r_{15})$$

$$* O + (H^+ + e^-) \underset{k_{16}^-}{\overset{k_{16}^+}{\rightleftharpoons}} * OH \quad (r_{16})$$

$$* OH + (H^+ + e^-) \underset{k_{17}^-}{\overset{k_{17}^+}{\rightleftharpoons}} * + H_2O \quad (r_{17})$$

$$* NO_2 + e^- \underset{k_{18}^-}{\overset{k_{18}^+}{\rightleftharpoons}} * + NO_2^- \quad (r_{18})$$

$$* NH_3 \underset{k_{19}^-}{\overset{k_{19}^+}{\rightleftharpoons}} * + NH_3 \quad (r_{19})$$

For the surface reactions, the rate constants for the forward and backward elementary reactions are determined by the Arrhenius equation:

$$k_i^+ = Ae^{-\frac{E_a}{k_bT}} \quad (S23)$$

$$k_i^- = Ae^{-\frac{E_b}{k_bT}} \quad (S24)$$

where k_i is the rate constant in s^{-1} for elementary step i, A is the pre-exponential factor, E_a is the activation energy, T is the temperature, and k_b is Boltzmann's constant. A was approximated as 10^{13} s^{-1} for all the elementary surface reactions[2]. For non-activated molecular adsorption, the rate of adsorption was determined by the rate of surface impingement of gas-phase molecules. Based on the Hertz-Knudsen equation[11], the molecular adsorption rate constant of species i was computed as:

$$k_{ads} = \frac{pA'}{\sqrt{2\pi m k_b T}} S \quad (S25)$$

where p is the partial pressure of the adsorbate in the gas phase, A' the surface area of the adsorption site, m the mass of the adsorbate, and S the sticking coefficient, which we assume takes a value of unity for all adsorbates. k_1^+, k_{18}^- and k_{19}^- should be calculated according to Eq. M12.

For molecular desorption, we assumed there are three rotational degrees of

freedom and two translational degrees of freedom in the activated state. Accordingly, the rate constant of desorption for adsorbate i was calculated as:

$$k_{des} = \frac{k_b T^3}{h^3} \frac{A'(2\pi k_b)}{\sigma \theta_{rot}} e^{-\frac{E_{des}}{k_b T}} \quad \text{(S26)}$$

where E_{des} is the desorption energy, h is Planck's constant, and σ and θ_{rot} are the symmetry number and the characteristic temperature for rotation, respectively. k_1^-, k_{18}^+ and k_{19}^+ should be calculated according to Eq. M13.

The nitrate reduction rate was calculated by the MKMCXX microkinetic modeling software suite for heterogeneous catalysis[3, 4]. In our simulations, the molar ratio of NO_3^- and H^+ in the solution was 1:1 at a reaction temperature of 300 K. For each of the M components in the kinetic network, a single differential equation for each elementary reaction step was written in the form of:

$$r_i = k_i \prod_{w=1}^{M} c_w^{v_w^i} \quad \text{(S27)}$$

where k_i is the rate constant and c_w and v_w^i are the concentration and stoichiometric coefficient of species w in elementary reaction step i. Specifically, all the involved constraints are as follows:

$$r_1 = k_1^+ P_{NO_3^-} \theta_* - k_1^- \theta_{*NO_3} \quad \text{(S28)}$$

$$r_3 = k_3^+ \theta_{*NO} P_{H^+} - k_3^- \theta_{*NOH} \quad \text{(S29)}$$

$$r_4 = k_4^+ \theta_{*NO} P_{H^+} - k_4^- \theta_{*NHO} \quad \text{(S30)}$$

$$r_5 = k_5^+ \theta_{*NOH} P_{H^+} - k_5^- \theta_{*NHOH} \quad \text{(S31)}$$

$$r_6 = k_6^+ \theta_{*NHO} P_{H^+} - k_6^- \theta_{*NHOH} \quad \text{(S32)}$$

$$r_7 = k_7^+ \theta_{*NHO} P_{H^+} - k_7^- \theta_{*NH_2O} \quad \text{(S33)}$$

$$r_8 = k_8^+ \theta_{*NHOH} P_{H^+} - k_8^- \theta_{*NH} P_{H_2O} \quad \text{(S34)}$$

$$r_9 = k_9^+ \theta_{*NHOH} P_{H^+} - k_9^- \theta_{*NH_2OH} \quad \text{(S35)}$$

$$r_{10} = k_{10}^+ \theta_{*NH_2O} P_{H^+} - k_{10}^- \theta_{*NH_2OH} \quad \text{(S36)}$$

$$r_{11} = k_{11}^+ \theta_{*NOH} P_{H^+} - k_{11}^- \theta_{*N} P_{H_2O} \quad \text{(S37)}$$

$$r_{12} = k_{12}^{+}\theta_{*N}P_{H^+} - k_{12}^{-}\theta_{*NH} \quad \text{(S38)}$$

$$r_{13} = k_{13}^{+}\theta_{*NH}P_{H^+} - k_{13}^{-}\theta_{*NH_2} \quad \text{(S39)}$$

$$r_{14} = k_{14}^{+}\theta_{*NH_2OH}P_{H^+} - k_{14}^{-}\theta_{*NH_2}P_{H_2O} \quad \text{(S40)}$$

$$r_{15} = k_{15}^{+}\theta_{*NH_2}P_{H^+} - k_{15}^{-}\theta_{*NH_3} \quad \text{(S41)}$$

$$r_{16} = k_{16}^{+}\theta_{*O}P_{H^+} - k_{16}^{-}\theta_{*OH} \quad \text{(S42)}$$

$$r_{17} = k_{17}^{+}\theta_{*OH}P_{H^+} - k_{17}^{-}\theta_{*}P_{H_2O} \quad \text{(S43)}$$

$$r_{18} = k_{18}^{+}\theta_{*NO_2} - k_{18}^{-}\theta_{*}P_{NO_2^-} \quad \text{(S44)}$$

$$r_{19} = k_{19}^{+}\theta_{*NH_3} - k_{19}^{-}\theta_{*}P_{NH_3} \quad \text{(S45)}$$

$$\theta_{*} + \theta_{*NO_3} + \theta_{*NO_2} + \theta_{*NO} + \theta_{*NHO} + \theta_{*NOH} + \theta_{*NHOH} + \theta_{*NH_2O} + \theta_{*N} + \theta_{*NH} + \theta_{*NH_2} + \theta_{*NH_3} + \theta_{*O} + \theta_{*OH} = 1 \quad \text{(S46)}$$

Steady-state coverages were computed by integrating the ordinary differential equations in time until changes in the surface coverages were small ($< 10^{-12}$). Rates of the individual elementary steps were obtained based on the computed steady-state surface coverages.

Supplementary Note S2. Details of Slow-growth method

Both the AIMD and Slow-growth approach simulations were sampled within the canonical (NVT) ensemble by Nosé-Hoover thermostats with a time step of 1.0 fs at a finite temperature of 300 K.

we mainly adopted the Slow-growth method[14, 15] to obtain the free energy barrier. In the Slow-growth method, a geometric parameter λ, i.e., the collective variable (CV), is constrained during the dynamics and varied from the value characteristic of the initial state (λ0) to that of the final state (λ1) with a velocity of transformation υ. The Helmholtz free energy difference ΔF can be computed as Equation (1) by collecting the derivative of the potential energy with respect to lambda during the simulation, and by integrating over lambda afterwards.

$$\Delta F = \int_{\lambda_0}^{\lambda_1} \left(\frac{\partial V(q)}{\partial \lambda}\right) \cdot \upsilon dt \quad \text{(S47)}$$

References for Supplementary Information.

1. Lide, D. R. CRC Handbook of Chemistry and Physics. CRC Press (2010).
2. van Santen, R. A., Markvoort A. J., Filot I. A. W., Ghouri M. M. Hensen E. J. M. Mechanism and microkinetics of the Fischer-Tropsch reaction. *Phys. Chem. Chem. Phys.* **15**, 17038-17063 (2013).
3. Filot, I. A. W., van Santen R. A. Hensen E. J. M. The Optimally Performing Fischer-Tropsch Catalyst. *Angew. Chem. Int. Ed.* **53**, 12746-12750 (2014).
4. Filot, I. A. W. et al. First-Principles-Based Microkinetics Simulations of Synthesis Gas Conversion on a Stepped Rhodium Surface. *ACS Catal.* **5**, 5453-5467 (2015).
5. Kim, K. H. et al. Energy-efficient electrochemical ammonia production from dilute nitrate solution. *Energy Environ. Sci.* **16**, 663-672 (2023).
6. He, W. H. et al. Splicing the active phases of copper/cobalt-based catalysts achieves high-rate tandem electroreduction of nitrate to ammonia. *Nat. Commun.* **13**, 1129 (2022).
7. Wang, Y. H. et al. Enhanced Nitrate-to-Ammonia Activity on Copper-Nickel Alloys via Tuning of Intermediate Adsorption. *J. Am. Chem. Soc.* **142**, 5702-5708 (2020).
8. Liu, H. M. et al. Efficient Electrochemical Nitrate Reduction to Ammonia with Copper-Supported Rhodium Cluster and Single-Atom Catalysts. *Angew. Chem. Int. Ed.* **61**, e202202556 (2022).
9. Wang, J. et al. Electrocatalytic Reduction of Nitrate to Ammonia on Low-Cost Ultrathin CoO_x Nanosheets. *ACS Catal.* **11**, 15135-15140 (2021).
10. Zhang, S., Li M., Li J. C., Song Q. N. Liu X. High-ammonia selective metal-organic framework-derived Co-doped Fe/Fe_2O_3 catalysts for electrochemical nitrate reduction. *Proc. Natl. Acad. Sci. U.S.A.* **119**, e2115504119 (2022).
